# FedREPA: Relation-Enhanced Prototype Alignment for Heterogeneous Federated Human Activity Recognition

**DOI:** 10.3390/s26217005

**Published:** 2026-11-05

**Authors:** Rongbo Zhou, Jiao Tian, Liejun Wang

**Affiliations:** School of Computer Science and Technology, Xinjiang University, Urumqi 830046, China; 107552503757@stu.xju.edu.cn

**Keywords:** federated learning, human activity recognition, non-IID data, prototype learning, contrastive learning, wearable sensors

## Abstract

Federated human activity recognition (HAR) with wearable sensors is challenged by label-skewed client data, missing local activities, and scarce mini-batch negatives. We propose FedREPA, a relation-guided framework that couples missing-class-aware relation consistency with prototype contrastive learning. Clients derive an inter-class relation prior from shared global class prototypes. A masked relation-consistency loss matches supported local relation rows to this prior. The same prior reweights negative classes in the prototype contrastive loss, giving greater emphasis to nearby prototypes when trust is held fixed. Global prototypes provide cross-client positive and negative anchors, including negatives for activities absent locally. On UCI HAR, PAMAP2, CIFAR-10, and CIFAR-100 under simulated Dirichlet label skew, FedREPA records the highest observed mean global test accuracy in the comparison with the ten evaluated baselines at each tested Dirichlet level, using the last five evaluation points per run. FedTGP, FedDPA, and FedAli-PHAR were not evaluated, and no performance advantage over them is claimed. Its paired accuracy margin over the restricted FedGPS-Core baseline averages about one percentage point. Worst-client accuracy is also highest among the methods shown on UCI HAR and CIFAR-10, while selected-class recall on CIFAR-10 shows mixed results. These findings are descriptive comparisons under the evaluated Dirichlet label-skew protocol. Performance under subject-wise federated partitions, device-wise heterogeneity, and sensor-placement variation remains unvalidated, as does clinical applicability.

## 1. Introduction

Human activity recognition (HAR) from wearable and mobile sensors enables continuous monitoring for healthcare, fitness, and smart environments [1,2,3,4,5]. Because sensor streams are privacy-sensitive and naturally distributed across users, federated learning (FL) has become an attractive training paradigm: clients update models locally and share only model information with a server, instead of uploading raw signals [6,7,8]. FedREPA does not share model parameters alone. A client also sends class prototypes, class counts, intra-class dispersions, and trust scores. The counts are that client’s activity histogram, which in healthcare or eldercare is itself sensitive; the prototypes and dispersions are class-conditional summaries of its features. The server is assumed honest-but-curious. Secure aggregation and differential privacy are possible directions for future work. Neither is implemented or experimentally evaluated in the reported runs, and no formal privacy guarantee or measured reduction in information exposure is established for the evaluated protocol. The contribution is relation-guided alignment under this information-sharing protocol. Section 5 discusses the associated disclosure risks. The federated formulation is motivated by on-device sensor applications, but the reported experiments are benchmark simulations rather than evaluations on smartphones, wearable hardware, or clinical systems.

In real HAR deployments, however, client data are not independent and identically distributed (non-IID) [9,10,11]. Different subjects perform different activities at different frequencies, so a client may observe only a subset of classes; local mini-batches then provide few (or no) negatives for discriminative learning. At the same time, semantically close activities are easily confused, and clients may form inconsistent class boundaries for the same activity set. Standard FedAvg [6] aggregates parameters by sample size and does not correct these semantic mismatches, which often leads to unstable optimization and reduced global accuracy under label skew. Our experiments vary client label distributions through Dirichlet partitioning. They do not construct clients by subject, device, or sensor placement, or separately evaluate these sources of heterogeneity.

Existing FL methods alleviate heterogeneity from complementary angles. Optimization-oriented approaches constrain local drift with proximal terms or control variates [12,13]. Representation-oriented approaches improve feature consistency through model-level contrast [14] or class prototypes shared across clients [15,16]. Sharpness-aware minimization (SAM) variants (e.g., FedSAM, MoFedSAM, and FedLESAM) further seek flatter minima for better generalization under client drift [17,18]. Prototype-based approaches differ in how they construct and use shared class references. Here, we study a specific use of their pairwise geometry: a shared inter-class relation prior for local relation consistency and class-dependent contrastive negative weighting.

We propose FedREPA (Federated Relation-Enhanced Prototype Alignment), a relation-guided FL framework for HAR under label skew. Clients derive an inter-class relation prior from global class prototypes. A masked relation-consistency loss matches supported local relation rows to the prior, while a prototype contrastive loss uses the same prior to reweight negative classes. Global prototypes provide cross-client positive and negative anchors even when local class coverage is incomplete. Relation information therefore directly shapes contrastive alignment. Trust-weighted aggregation is optional. The semantic SAM branch is documented for reproducibility, but its reported head-only configuration introduces no effective perturbation and has no demonstrated accuracy benefit.

For example, one client may observe only Sitting and Walking, while another observes Standing and Walking Upstairs. Neither client observes Sitting and Standing together, although these postures may be confused. The clients also learn ambulation representations from different local class sets. FedREPA uses global prototypes as shared class anchors and the relation prior to increase the relative emphasis on nearby negative classes.

The main contributions of this paper are as follows:

- We propose FedREPA for federated HAR under Dirichlet label skew. Its methodological contribution is the joint use of a prototype-derived inter-class relation prior for missing-class-aware relation consistency and class-dependent negative weighting in prototype contrastive learning. The contribution lies in how these objectives use the same prior, rather than in introducing consistency regularization or feature alignment as general ideas.
- We match local and global relation distributions using a masked Jensen–Shannon loss. Only classes present in the current mini-batch and supported by sufficient local samples contribute rows to the relation loss; the global prototypes of locally missing classes remain available as contrastive negatives.
- We design a relation-enhanced prototype contrastive objective that uses global prototypes as cross-client positive and negative anchors and reweights hard negatives by relation proximity. Semantic SAM is not required to define or optimize this objective together with relation consistency and cross-entropy. Its perturbation is zero in the reported head-only configuration, so it is not claimed as a validated contributor to the observed performance.
- Experiments on UCI HAR, PAMAP2, CIFAR-10, and CIFAR-100 give FedREPA the highest observed mean global accuracy in the comparison with the ten evaluated baselines at each tested Dirichlet level. All 48 matched per-seed accuracy differences against FedGPS-Core are positive, with mean +1.01 and standard deviation 0.19 percentage points; these differences are descriptive, not independent confirmations of superiority. The auxiliary results are metric-specific: worst-client accuracy is highest among the methods shown on UCI HAR and CIFAR-10, whereas selected-class recall is not uniformly higher on CIFAR-10.

These comparisons are limited to the evaluated Dirichlet label-skew settings; Section 4.2 reports their statistical limitations. FedTGP, FedDPA, and FedAli-PHAR are discussed but not evaluated, so these results do not establish an advantage over those methods. FedGPS-Core retains the prototype and update-direction cues of FedGPS but drops its client-side surrogate path and aligns prototypes on real local features. The reported margin therefore concerns this restricted adaptation under our information-sharing protocol, not published FedGPS.

The remainder of this paper is organized as follows. Section 2 reviews related work. Section 3 presents the problem setup and FedREPA. Section 4 reports the experimental results. Section 5 discusses the findings and limitations. Section 6 concludes the paper.

## 2. Related Work

We organize related work by the strategy used to address non-IID data in federated learning, emphasizing what existing methods optimize and what gaps remain for relation-guided prototype alignment.

### 2.1. Optimization-Based Correction of Client Drift

FedAvg aggregates local updates by sample size and serves as the standard baseline for FL [6,7]. Under label skew, however, local objectives diverge, and client drift degrades the global model [9]. FedProx adds a proximal term to keep local parameters close to the global model [12], while SCAFFOLD uses control variates to correct update bias [13]. More recent work such as FedGPS rectifies statistical heterogeneity by coupling goal-level distribution modeling with path-level gradient synergy, and it achieves strong performance under label skew [19]. However, FedGPS does not explicitly encode inter-class semantic relations as contrastive guidance, and its original design uses a client-side surrogate set whose prototypes are aggregated by the server, which is outside the messages exchanged by the other methods in this study. Our experiments instead evaluate FedGPS-Core, a restricted adaptation that omits the client-side surrogate path and aligns prototypes on real local features, as defined in Section 4. These optimization-based methods mainly stabilize parameter aggregation; they do not use inter-class relation structure to reshape local representation learning.

### 2.2. Prototype-Based Semantic Alignment

Prototype-based FL communicates class-wise feature means as compact semantic summaries, following the idea of class prototypes in metric learning [20]. FedProto aligns local prototypes to global prototypes with a regularization term [15]. FedNH improves prototype geometry on the hypersphere and injects class semantics to mitigate imbalance-induced collapse [16].

FedTGP learns global prototypes on the server using adaptive-margin contrastive optimization [21]. FedDPA combines adaptive local-to-global prototype regularization, hierarchical aggregation, and contrastive alignment of class prototypes [22]. FedAli-PHAR uses optimal transport to align embeddings with learnable local and global prototypes for personalized HAR [23]. Prototype-based fine-tuning instead corrects heterogeneous classifiers after aggregation [24].

FedREPA’s specific design is to derive a row-wise inter-class relation distribution $\pi_{g}$ from global prototypes and use it in two client-side objectives: as the target of masked relation consistency in (4), and as class-pair-dependent negative weights in (5) and (6). The distinction concerns how and where the relation information enters the objective; it is not a claim that other prototype methods ignore inter-class geometry. The missing-class mask affects only the relation-consistency loss, while the global prototypes of locally absent classes remain contrastive negatives. These are methodological distinctions, not evidence of higher accuracy, robustness, or efficiency than FedTGP, FedDPA, or FedAli-PHAR. None of these three methods is included in the experimental comparison.

### 2.3. Contrastive Representation Learning in FL

Contrastive objectives improve feature discriminability by pulling positive pairs together and pushing negatives apart [25,26]. MOON applies model-level contrast between local and global representations to reduce representation drift [14]. However, MOON does not exploit class prototypes as cross-client negatives, and it does not differentiate easy versus hard negatives using a global relation structure. When local mini-batches contain few classes, batch-internal negatives remain scarce. FedREPA addresses this gap by using global prototypes as positive and negative anchors and by reweighting negatives according to relation proximity, so semantically close activities receive stronger separation pressure.

### 2.4. Sharpness-Aware Federated Optimization

Sharpness-aware minimization (SAM) seeks parameters in flat loss neighborhoods and improves generalization in centralized training [27]. Federated variants adapt this idea to client drift: FedSAM and MoFedSAM introduce SAM-based local updates and momentum coupling with global directions within one framework [17], while FedLESAM estimates global perturbation directions more efficiently from consecutive global models [18], and later variants extend the idea to decentralized topologies with privacy constraints [28]. These optimizers improve the geometry of local minima, but they do not supply missing semantic negatives or encode category relations. SAM-style optimization is a possible extension of prototype-based alignment. The reported FedREPA configuration, however, applies no semantic perturbation and does not demonstrate a benefit from semantic sharpness-aware optimization.

### 2.5. Federated Learning for Sensor-Based HAR

HAR on wearable and mobile sensors is a natural FL application because activity data are privacy-sensitive and distributed across users [1,2]. Early studies use federated training for HAR models based on on-device inertial data [29], while FedHealth transfers wearable representations across users [30]. ClusterFL exploits similarity-aware clustering to improve accuracy and communication efficiency for HAR [31], and FedAKD enables heterogeneous on-device models through augmented knowledge distillation [32]. Recent federated HAR studies address heterogeneity from four complementary directions: aligning the raw signal space on device before training [33], clustering clients dynamically for personalization [34], combining contrastive learning with feature distillation [35], and compressing personalized models for resource-constrained wearables [36]. Closest to our setting, FedAli-PHAR aligns embeddings to learnable local and global prototypes through optimal transport [23]. FedREPA focuses on using prototype-derived inter-class relations in masked consistency and local contrastive negative weighting. This objective differs from optimal-transport embedding alignment, but that design distinction does not establish an accuracy or robustness advantage over FedAli-PHAR.

### 2.6. Positioning

Table 1 distinguishes the availability of cross-client class anchors, the treatment of negative classes, and the explicit handling of missing local classes. FedREPA uses one supervised local model per client and exchanges model updates and class statistics with a server; it does not use reciprocal pseudo-label supervision between peer networks. Its relation-consistency loss matches distributions of inter-class prototype similarities, rather than requiring agreement between sample-wise predictions of peer models or perturbed views. Its prototype contrastive loss aligns sample features with global class anchors while allowing the negative weight to depend on both the sample label and the negative class.

The specific distinction from prototype alignment alone is where the relation information enters the objective. The global relation prior $\pi_{g}$ supplies a target for the masked loss $L_{\mathrm{rel}}$ and, through $w_{c}\left(y_{i}\right)$, determines the class-pair-dependent weights inside the denominator of $L_{\mathrm{pcon}}$. The mask prevents unsupported local classes from contributing relation rows; it does not remove their global prototypes from the contrastive negative set. This coupling of structural matching and discriminative class separation is the claimed methodological contribution. It does not establish an empirical advantage over cross-teaching or other consistency-based methods outside the evaluated baselines.

In Table 1, FedGPS is the published method. FedGPS-Core, used in the experiments, is the restricted adaptation defined in Section 4. FedTGP and FedAli-PHAR appear only in this qualitative comparison, and FedDPA is discussed in the text; none of the three is an evaluated baseline. The FedAli-PHAR entry records its transport-based assignment mechanism without assuming uniform contrastive negative weights.

## 3. Proposed Method

### 3.1. Problem Setup

We consider a *C*-class recognition problem trained by *K* clients under the standard federated averaging protocol [6]. Client *k* holds a private labeled set $D_{k}=\left\{\left(x_{i},y_{i}\right)\right\}$ with $n_{k}=\left|D_{k}\right|$ samples. A shared model consists of an encoder ϕ with parameters *w* and a linear classifier. For an input *x*, the encoder produces a feature $z=\phi \left(x\right)\in R^{d}$ that is $\ell_{2}$-normalized onto the unit sphere; cosine similarity is then $\mathrm{sim}\left(u,v\right)=u^{⊤}v$. The local supervised loss is the cross-entropy $L_{\mathrm{CE}}$ on classifier logits.

Training proceeds in communication rounds t=1,…,T. In round *t*, the server samples a subset $S^{t}$ of clients, broadcasts the current global parameters $w_{g}^{t}$, and each selected client performs local stochastic gradient descent (SGD) steps before returning an update. The server aggregates the received updates into $w_{g}^{t+1}$. Under label skew, $D_{k}$ typically covers only a subset of the *C* activities: a mini-batch may contain few negatives, and the same activity (e.g., Walking) can be paired with different neighbors on different clients. All prototype and relation computations below are performed on the unit sphere, so they remain comparable across clients with different feature scales.

Let $p_{k,c}$ denote the local prototype of class *c* on client *k*, defined as the normalized mean of encoder features of that class, and let $P^{g}={\left\{p_{c}^{g}\right\}}_{c=1}^{C}$ denote the server-side global prototypes. We write $n_{k,c}$ for the number of local samples of class *c*. A missing-class mask $m_{c}\in \left\{0,1\right\}$ is set to $m_{c}=1$ if class *c* appears in the current mini-batch *and* client *k* holds at least $n_{\min}$ samples of that class; otherwise $m_{c}=0$. The remaining symbols are listed in Table 2.

FedREPA derives a global relation prior $\pi_{g}$ from the geometry of shared class prototypes. We use three terms consistently: prototype alignment refers to the use of global prototypes as class anchors in $L_{\mathrm{pcon}}$; the relation prior is $\pi_{g}$; and trust weighting refers to the use of trust scores in aggregation and, when enabled, in negative weights. FedGPS denotes the published method, whereas FedGPS-Core denotes the restricted experimental baseline. The relation prior directly determines contrastive negative weights rather than acting only as a separate regularizer. Trust-weighted aggregation remains optional. The semantic SAM implementation is retained in the description of the reported runs, with its zero perturbation and unvalidated benefit stated explicitly.

### 3.2. Overview

**Figure 1:**
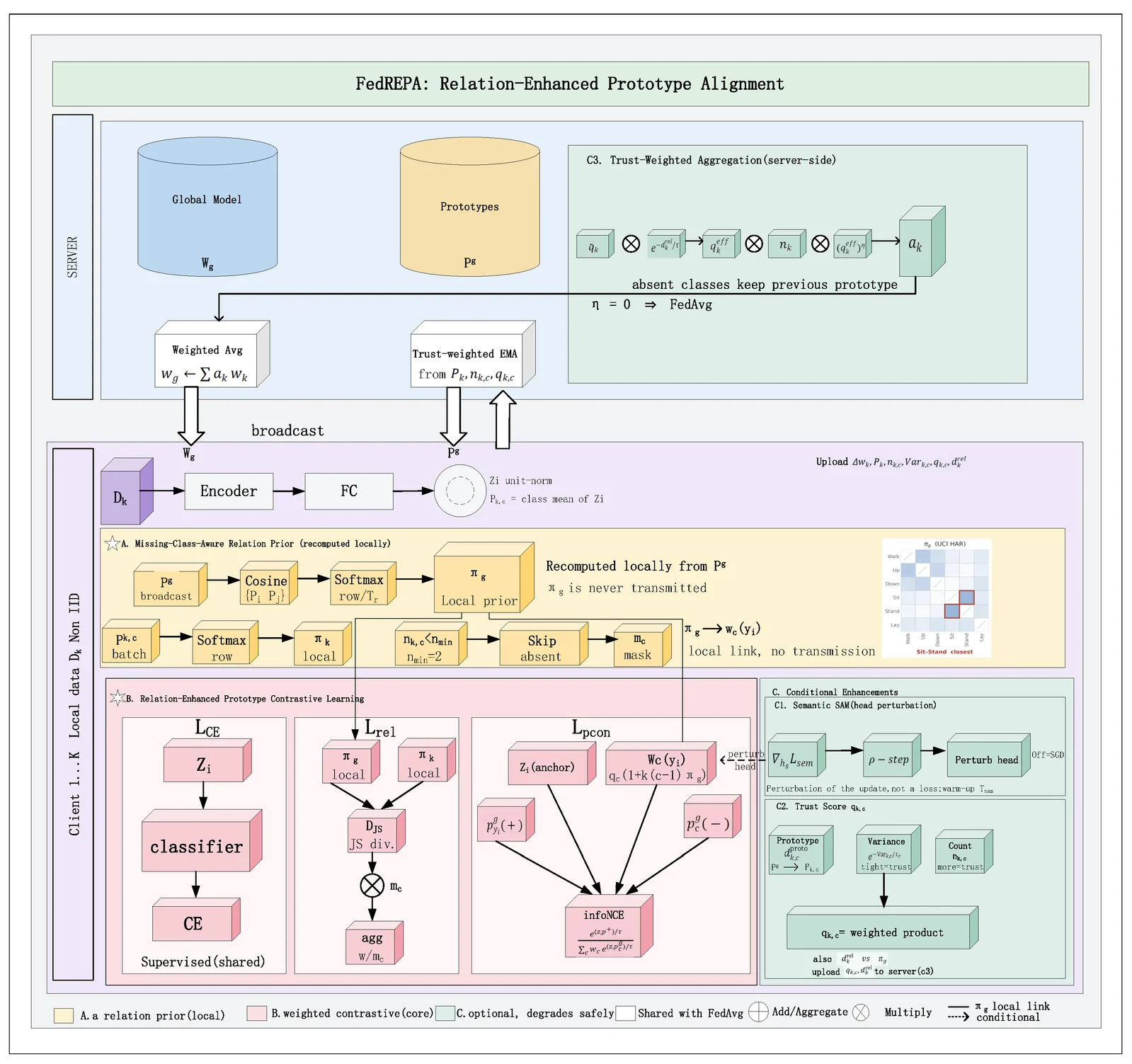
Overview of FedREPA in one communication round. The server broadcasts the model $w_{g}$, prototypes $P^{g}$, and class trust $q^{g}$ when enabled. (**A**) Each client recomputes $\pi_{g}$ using a row-wise softmax of global-prototype cosine similarities; $\pi_{g}$ is not transmitted. The local mask $m_{c}$ applies only to relation-loss rows: class *c* must occur in the current mini-batch and have at least $n_{\min}=2$ local samples. The mask does not restrict prior construction or contrastive negatives. The UCI HAR heatmap highlights the Sitting–Standing pair. (**B**) $L_{\mathrm{rel}}$ matches $\pi_{k}$ to $\pi_{g}$ using masked JS divergence, and $L_{\mathrm{pcon}}$ uses $\pi_{g}$ to reweight prototype negatives, including locally absent classes. Both terms enter the local objective with cross-entropy. (**C1**) The conditional semantic SAM path is shown schematically; in the reported head-only setting, the semantic gradient is zero, and the cosine gate adds no perturbation, while SGD on the full objective still updates the classifier. (**C2**) Clients compute trust and relation deviation. (**C3**) The server forms aggregation weights from uploaded updates and class statistics; η=0 recovers FedAvg model aggregation. After training, clients upload $\Delta w_{k}$, $P_{k}$, $n_{k,c}$, ${\mathrm{Var}}_{k,c}$, $q_{k,c}$, and $d_{k}^{\mathrm{rel}}$.

Figure 1 shows one communication round of FedREPA.

Each round proceeds as follows. The server broadcasts $w_{g}$ and $P^{g}$ to the selected clients, together with the class-trust vector $q^{g}$ when the trust module is on. Client *k* recomputes $\pi_{g}$ from $P^{g}$, extracts normalized features, forms a local relation estimate, and minimizes

(1) $$L_{\mathrm{base}}^{k}\left(w\right)=L_{\mathrm{CE}}^{k}\left(w\right)+\lambda_{1}L_{\mathrm{rel}}^{k}\left(w\right)+\lambda_{2}L_{\mathrm{pcon}}^{k}\left(w\right).$$

The two auxiliary losses act at different levels of the representation. Relation consistency matches class-to-class relation distributions and encourages supported local class geometry to agree with the shared global prior. Prototype contrastive learning acts on individual samples, aligning their features with the correct global class anchor and separating them from competing anchors. Their weighted gradients are combined with supervised cross-entropy to train the client’s encoder.

After local training, the client uploads the parameter update, its local prototypes $P_{k}$, the class counts $n_{k,c}$, the intra-class dispersions ${\mathrm{Var}}_{k,c}$, the per-class trust scores $q_{k,c}$ of (7), and a scalar relation deviation $d_{k}^{\mathrm{rel}}$. To an honest-but-curious server, $n_{k,c}$ is the client’s activity histogram, while $P_{k}$ and ${\mathrm{Var}}_{k,c}$ are first- and second-moment summaries of its features. Trust is computed on the client from $P_{k}$, ${\mathrm{Var}}_{k,c}$, $n_{k,c}$, and the broadcast $P^{g}$. Because these statistics are uploaded, the server can recompute (7) without accessing raw sensor windows. This access to individual client statistics is part of the evaluated information-sharing protocol. The trust scores serve as optimization weights and do not provide a privacy guarantee. The server aggregates parameters and updates $P^{g}$ by a count-and-trust-weighted exponential moving average (EMA). Clients recompute $\pi_{g}$ from the new $P^{g}$ in the next round. Algorithm 1 summarizes the communication round. The following subsections define the two core losses and the optional modules.
**Algorithm 1** FedREPA, one communication round *t***Require:** 
global model $w_{g}$, prototypes $P^{g}$ (random unit vectors at t=1), class trust $q^{g}$, warm-up lengths $T_{\mathrm{rel}},T_{\mathrm{sam}}$  1:sample a client subset $S^{t}$ and broadcast $\left(w_{g},P^{g},q^{g}\right)$  2:**for all** clients $k\in S^{t}$ **in parallel do**  3:      $w_{k}\leftarrow w_{g}$; $\pi_{g}\leftarrow$ row-normalized prior from $P^{g}$ by (2)        ▷ local; never uploaded  4:      clear the relation cache ${\hat{\pi }}_{k}$  5:      **for** each local mini-batch *B* **do**  6:            z←$\ell_{2}$-normalized encoder features of *B*  7:            form batch prototypes and $\pi_{k}^{\mathrm{batch}}$; set the mask $m_{c}$ from the classes present in *B*  8:            ${\hat{\pi }}_{k}\leftarrow \pi_{k}^{\mathrm{batch}}$ on the first batch, else EMA update; blend to $\pi_{k}$ by (3)  9:            $L\leftarrow L_{\mathrm{CE}}+\lambda_{2}L_{\mathrm{pcon}}$ with relation-weighted negatives (5), (6)10:            **if** $t>T_{\mathrm{rel}}$ **then**11:                  $L\leftarrow L+\lambda_{1}L_{\mathrm{rel}}$ by (4)                                                   ▷$L_{\mathrm{pcon}}$ has no warm-up12:            **end if**13:            **if** $t>T_{\mathrm{sam}}$ **and** semantic SAM enabled **then**14:                  back-propagate the semantic loss; the extracted head gradient and gated perturbation are zero15:                  clear preliminary gradients; recompute *L* and its gradient at unperturbed weights, including the relation-cache update16:            **end if**17:            take an SGD step on *L*18:      **end for**19:      compute $P_{k}$, $n_{k,c}$, ${\mathrm{Var}}_{k,c}$, $q_{k,c}$ (7) and $d_{k}^{\mathrm{rel}}$ (9); upload with $w_{k}-w_{g}$20:**end for**21:$w_{g}\leftarrow \sum_{k\in S^{t}}a_{k}w_{k}$ with $a_{k}$ from (10)                                            ▷η=0 recovers FedAvg22:update $P^{g}$ and $q^{g}$ by count-and-trust-weighted EMA; classes reported by no client keep their previous value

Global prototypes are initialized as shared random unit vectors, so $\pi_{g}$ is defined from the first round. Only $L_{\mathrm{rel}}$ is delayed until after the warm-up $T_{\mathrm{rel}}$; $L_{\mathrm{pcon}}$ is active from round t=1 to provide negatives when local batches contain few classes. Thus, the contrastive term initially pulls features toward anchors with little semantic information. With μ=0.5, each reported prototype is updated in every round, including the first, by mixing its previous vector with the uploaded class mean and normalizing the result. The random component therefore decays over rounds in which the class is reported rather than disappearing after round t=1. An unreported class retains its initial vector until a selected client reports it. All clients receive the same anchors, so the initial targets are shared rather than client-specific. At $T_{r}=1.0$, the cosine similarities between random unit vectors are near zero, making the initial relation weights in (5) approximately uniform. Delaying $L_{\mathrm{rel}}$ avoids matching this initially uninformative structure; it does not delay the entire semantic pipeline. The duration of the initial anchors’ influence was not measured. Cross-entropy does not directly depend on $P^{g}$, although the auxiliary losses affect the training trajectory.

The implementation follows the sequence below. (1) At t=1, every global prototype is an independent random unit vector. At the end of the round, after $w_{g}$ has been aggregated, each reported class is updated by $p_{c}^{g}\leftarrow \mathrm{normalize}\left(\mu p_{c}^{g}+\left(1-\mu \right){\bar{p}}_{c}\right)$ with μ=0.5, where ${\bar{p}}_{c}$ is the count-and-trust-weighted mean of the uploaded prototypes; a class reported by no selected client keeps its previous vector. (2) On client *k*, class *c* is treated as missing when the client holds fewer than $n_{\min}=2$ of its samples. The mask bit is then zero; that row is omitted from $L_{\mathrm{rel}}$, and an uploaded prototype with count zero does not enter the update of $p_{c}^{g}$. Inside a mini-batch, a class that is absent from the batch uses the detached $p_{c}^{g}$ only as a relation anchor. (3) The relation cache ${\hat{\pi }}_{k}$ is cleared when the round starts. The first mini-batch that contains class *i* copies $\pi_{k}^{\mathrm{batch}}\left(i,:\right)$ into the cache and does not blend; every later batch in that round applies (3) with stop-gradient through ${\hat{\pi }}_{k}$. Rows not yet seen stay untouched. (4) After the local epochs, one forward pass on the updated model produces $P_{k}$, $n_{k,c}$, and ${\mathrm{Var}}_{k,c}$, and $q_{k,c}$ is computed using (7). The server sets $q_{c}^{g}$ by an EMA, with coefficient 0.9, of the count-weighted average of the uploaded scores. Every class begins at $q_{c}^{g}=0.5$, and an unreported class keeps its previous value. (5) The aggregation weight is (10), using $n_{k}$ and $q_{k}^{\mathrm{eff}}=q_{k}\exp\left(-d_{k}^{\mathrm{rel}}/\tau_{\mathrm{dev}}\right)$. Setting η=0 removes the trust factor and recovers FedAvg. (6) Semantic SAM, on every second local step after round $T_{\mathrm{sam}}$, back-propagates $\lambda_{1}L_{\mathrm{rel}}+\lambda_{2}L_{\mathrm{pcon}}$ and restricts the perturbation to parameters named head, the output classifier. Those two losses do not depend on head, so that gradient is zero. The cosine gate then contributes no non-self perturbation. No semantic perturbation is applied. The preliminary gradients are cleared before a second forward/backward pass on the full local objective. The optimizer updates the model at unperturbed weights. Both loss evaluations update the relation cache when relation consistency is active; this repeated evaluation is an implementation effect, not a nonzero SAM perturbation.

### 3.3. Missing-Class-Aware Global Relation Prior

From the broadcast prototypes $P^{g}$, each client forms a pairwise relation matrix and a row-wise distribution

(2) $$R_{g}\left(i,j\right)=\mathrm{sim}\left(p_{i}^{g},p_{j}^{g}\right),\;\pi_{g}\left(i,j\right)=\frac{\exp(R_{g}\left(i,j\right)/T_{r})}{\sum_{l\neq i}\exp\left(R_{g}\left(i,l\right)/T_{r}\right)},\;j\neq i,$$

where $T_{r}$ is a temperature. The *i*-th row of $\pi_{g}$ is a distribution over the other C−1 classes and encodes which categories are close to class *i*. Jensen–Shannon (JS) divergence [37] is used later to *match* local rows to $\pi_{g}$; it is not used to construct $\pi_{g}$.

On client *k*, a differentiable local prototype is formed from the current mini-batch: if class *c* appears, $p_{k,c}^{\mathrm{batch}}$ is the normalized mean of its batch features; otherwise the detached global prototype $p_{c}^{g}$ is used only as a relation anchor and the corresponding row is masked out. Applying (2) to these prototypes yields a batch-level distribution $\pi_{k}^{\mathrm{batch}}$. Under strong label skew, a mini-batch covers few classes, so the client maintains a stop-gradient EMA cache ${\hat{\pi }}_{k}$ within the round and blends

(3) $$\pi_{k}\left(i,:\right)=\left(1-\beta_{\mathrm{rel}}\right)\;\pi_{k}^{\mathrm{batch}}\left(i,:\right)+\beta_{\mathrm{rel}}\;{\hat{\pi }}_{k}\left(i,:\right).$$

Gradients flow only through the current-batch term. The cache is cleared at the beginning of each round and seeded by the first mini-batch that contains a given class, so (3) is an identity on that batch and the blend only takes effect from the second occurrence onward; rows for classes not yet seen in the round are left untouched rather than being filled with a uniform or global value. The cache is cleared because aggregation changes the encoder, which can make estimates from the previous round stale.

Let $q_{c}^{g}$ be an optional class-wise trust score (Section 3.5; it may be set uniformly to 1). With mask $m_{i}$ and row-wise JS divergence $D_{\mathrm{JS}}$, the relation-consistency loss is

(4) $$L_{\mathrm{rel}}^{k}=\frac{\sum_{i=1}^{C}m_{i}\;q_{i}^{g}\;D_{\mathrm{JS}}\left(\pi_{g}\left(i,:\right)\;∥\;\pi_{k}\left(i,:\right)\right)}{\sum_{i=1}^{C}m_{i}\;q_{i}^{g}+\varepsilon }.$$

Relation consistency provides a structural regularizer on local class representatives. For each supported class, it matches the distribution of similarities to the other classes to the corresponding row of the global prior. Gradients reach the encoder through the current-batch class means. Only supported rows contribute matching terms; detached global prototypes can still represent absent classes as anchors within those rows. This loss therefore encourages agreement in relative class structure across clients. It does not directly classify individual samples or explicitly contrast each sample with its positive and negative class anchors.

### 3.4. Relation-Enhanced Prototype Contrastive Learning

Local mini-batches under skew provide too few negatives, and treating every negative class equally ignores that some activities are semantically close and more likely to be confused. On UCI HAR, the two static postures Sitting and Standing are a close pair, as is the ambulation triple Walking/Walking Upstairs/Walking Downstairs. FedREPA therefore uses the global prototypes as cross-client positives and negatives, and reweights negatives by relation proximity.

For a sample with feature $z_{i}$ and label $y_{i}$, the positive is $p_{y_{i}}^{g}$ and every $c\neq y_{i}$ is a negative. Let ${\tilde{q}}_{c}=\max\left(q_{c}^{g},q_{\min}\right)$ be a lower-bounded trust score. The negative weight is

(5) $$w_{c}\left(y_{i}\right)={\tilde{q}}_{c}\left(1+\kappa \left(C-1\right)\pi_{g}\left(y_{i},c\right)\right),\;c\neq y_{i}.$$

The factor (C−1) makes κ an interpretable relative strength, because each row of $\pi_{g}$ averages 1/(C−1) off the diagonal: κ=0 removes relation weighting and keeps only the trust gate, whereas ${\tilde{q}}_{c}=1$ removes the gate and keeps a purely relation-aware contrast. The prototype contrastive loss is

(6) $$L_{\mathrm{pcon}}^{k}=-\frac{1}{B}\sum_{i=1}^{B}\log\frac{\exp(\mathrm{sim}\left(z_{i},p_{y_{i}}^{g}\right)/\tau )}{\exp\left(\mathrm{sim}\left(z_{i},p_{y_{i}}^{g}\right)/\tau \right)+\sum_{c\neq y_{i}}w_{c}\left(y_{i}\right)\exp\left(\mathrm{sim}\left(z_{i},p_{c}^{g}\right)/\tau \right)},$$

where τ is a contrastive temperature. It may be a shared constant or class-wise adaptive; the choice is selected on a held-out validation split and is reported in Section 4. The prototype contrastive loss provides sample-level discrimination. Each feature is trained to increase its similarity to the global prototype of its label relative to competing class prototypes. Global prototypes provide negative anchors even when the corresponding classes are absent from the client or the current mini-batch. The relation prior determines the negative weights: when trust is held fixed, a larger $\pi_{g}\left(y_{i},c\right)$ gives class *c* a larger weight in the denominator. Thus, global prototypes address the shortage of local negatives, while relation weighting determines their relative emphasis. The missing-class mask acts only in $L_{\mathrm{rel}}$; it neither modifies $\pi_{g}$ nor removes locally absent classes from the negatives in $L_{\mathrm{pcon}}$. We do not add a separate prototype-classification term, whose positive part duplicates the numerator of (6).

The losses share the client’s encoder and the same global relation prior, but neither is a preprocessing stage for the other. The prior is computed directly from the broadcast global prototypes. With all other settings fixed, setting $\lambda_{1}=0$ removes explicit relation-distribution matching while retaining relation-weighted prototype contrastive learning when $\lambda_{2}>0$ and κ>0. Setting $\lambda_{2}=0$ removes the sample-to-prototype contrastive term while retaining relation consistency when $\lambda_{1}>0$ and its warm-up is complete. Cross-entropy remains active in both cases. Setting κ=0 instead removes the relation-dependent factor from the negative weights; the prototype contrastive objective and its trust weighting remain, as does relation consistency when enabled. Structural consistency and sample-level discrimination are therefore distinct uses of the same prior.

On UCI HAR, the learned relation prior identifies two semantically close class pairs: Sitting–Standing (the closest prototype pair, $R_{g}=0.69$) and Walking–Walking Upstairs ($R_{g}=0.68$). In (5), a larger relation probability increases a negative class’s weight when trust is held fixed. With $T_{r}=1$, the rows of $\pi_{g}$ remain fairly flat, so the induced weights span roughly 1.7–2.3 rather than an order of magnitude; $T_{r}$ therefore controls how aggressive the hard-negative emphasis becomes, and smaller values sharpen it. The main tables use κ=1.0 and $T_{r}=1.0$. A separate UCI HAR sweep, reported in Section 5, varies one coordinate at a time around that point. The weight range above describes $T_{r}=1$; it is not itself that sweep.

### 3.5. Conditional Enhancements

The modules below are optional. They can be disabled independently; the remaining method reduces to (1) with FedAvg aggregation.

#### 3.5.1. Class-Wise Trust

A compact quality score combines local–global prototype agreement, intra-class dispersion, and sample support:

(7) $$q_{k,c}=\exp\;\left(-\frac{∥p_{k,c}-p_{c}^{g}{∥}_{2}^{2}}{\tau_{d}}\right)\cdot \exp\;\left(-\frac{{\mathrm{Var}}_{k,c}}{\tau_{v}}\right)\cdot \sqrt{\frac{n_{k,c}}{n_{k,c}+n_{0}}},$$

where ${\mathrm{Var}}_{k,c}=\frac{1}{n_{k,c}}\sum_{i:y_{i}=c}{∥z_{i}-p_{k,c}∥}_{2}^{2}$ is the intra-class dispersion of class *c* on client *k*, and $\tau_{d},\tau_{v},n_{0}$ are scale constants. The server aggregates $\left\{q_{k,c}\right\}$ by sample count and applies an EMA to obtain $q_{c}^{g}$. Classes not reported in the current round keep their previous value. The client-level score used below is the support-weighted mean $q_{k}=\sum_{c}n_{k,c}q_{k,c}/\sum_{c}n_{k,c}$. This score may weight (4) and (5); it is a reliability gate, not a required source of accuracy gain.

#### 3.5.2. Semantic SAM

Sharpness-aware minimization (SAM) is a perturbation of the update, not an extra additive loss [17,27]. The semantic SAM module defines its perturbation direction from the semantic sub-loss rather than the full objective,

(8) $$g_{k}^{\mathrm{sem}}=\nabla_{w}\left(\lambda_{1}L_{\mathrm{rel}}^{k}+\lambda_{2}L_{\mathrm{pcon}}^{k}\right).$$

The selected perturbation parameters in the reported runs are those named head, the output classifier that maps features to logits. The feature extractor and projection are outside this selected set. Since $L_{\mathrm{rel}}$ and $L_{\mathrm{pcon}}$ depend on encoder features and prototypes rather than classifier logits, their gradient on head is zero. For a nonzero non-self direction d, the enabled conflict gate uses g·d/((∥g∥+ ε)∥d∥) with $\varepsilon =10^{-8}$. With g=0, this quotient and the gate coefficient are zero. If the non-self direction is absent or has norm below ε, the implementation uses the zero local direction directly. The resulting perturbation is therefore zero in either case, regardless of the configured radius or its class-wise scaling.

Consequently, semantic SAM contributes no effective parameter perturbation in the evaluated head-only configuration and is not necessary for optimizing the core relation-alignment objective. The preliminary semantic gradients are cleared, and the optimizer uses the gradient of the full local loss at unperturbed weights. Cross-entropy updates the classifier, while the full loss trains the encoder. We retain the SAM branch in the implementation description to document how the reported runs were executed, not as a validated source of accuracy or generalization gains. The reported results establish neither the benefit nor the necessity of semantic sharpness-aware optimization.

Zero parameter perturbation does not imply that disabling the entire implementation branch reproduces an identical training trajectory. The additional semantic forward pass evaluates the relation loss and updates its within-round cache; the subsequent full-loss pass evaluates it again. Disabling the branch removes this extra evaluation and cache update as well as the preliminary backward pass. These implementation effects have not been isolated experimentally and are not evidence of a sharpness-aware benefit. A test of an effective semantic perturbation would require parameters on which the semantic loss has a nonzero gradient and a matched comparison controlling the loss-evaluation and cache-update schedule.

#### 3.5.3. Trust-Weighted Aggregation

After local training, client *k* reports a scalar structural deviation

(9) $$d_{k}^{\mathrm{rel}}=\frac{1}{|C_{k}|}\sum_{i\in C_{k}}D_{\mathrm{JS}}\left(\pi_{g}\left(i,:\right)\;∥\;\pi_{k}\left(i,:\right)\right),$$

where $C_{k}$ is the set of classes actually held by the client. The server forms an effective trust $q_{k}^{\mathrm{eff}}=q_{k}\exp\left(-d_{k}^{\mathrm{rel}}/\tau_{\mathrm{dev}}\right)$ and aggregates with

(10) $$a_{k}=\frac{n_{k}\;{\left(q_{k}^{\mathrm{eff}}\right)}^{\eta }}{\sum_{j\in S^{t}}n_{j}\;{\left(q_{j}^{\mathrm{eff}}\right)}^{\eta }}.$$

The case η=0 recovers FedAvg. Global prototypes are then updated by a trust-weighted EMA over the uploaded $\left\{p_{k,c}\right\}$; absent classes keep the previous prototype.

### 3.6. Communication and Computational Overhead

For the reported backbone configurations, counting the network parameters gives 120,710 for UCI HAR (SensorCNN), 141,132 for PAMAP2 (SensorCNN), 1,756,682 for CIFAR-10 (SimpleCNN), and 11,220,132 for CIFAR-100 (ResNet-18 with GroupNorm). At four bytes per parameter, their model weights occupy approximately 0.460, 0.538, 6.701, and 42.801 MiB, respectively (1 MiB $=2^{20}$ bytes). These counts include the feature extractor and classifier. Methods within a dataset share the same backbone dimensions; algorithm-specific classifier rules and frozen parameters may differ. These are network-parameter counts and weight-storage estimates, not peak graphics processing unit (GPU) memory measurements. Gradients, activations, optimizer state, client-model replicas, and method-specific auxiliary tensors require additional memory.

FedREPA uses the encoder and output classifier for prediction. Prototype estimation, relation matching, trust aggregation, and the additional semantic backward pass belong to training and are not required for a prediction with the trained global model. The relation prior therefore adds no trainable inference module to the shared backbone. This describes the inference computation; it is not a measured inference-latency result.

Relative to FedAvg, FedREPA additionally communicates class prototypes and statistics. A participating client’s prototype-and-statistic upload contains Cd+3C+1 floating-point entries: Cd prototype coordinates, *C* counts, *C* dispersions, *C* trust scores, and one relation-deviation scalar. In fp32, this is approximately 4.57 KiB on UCI HAR (C=6, d=192) and 9.14 KiB on PAMAP2 (C=12, d=192), or 0.97% and 1.66% of the corresponding model-weight payload (1 KiB $=2^{10}$ bytes). The downlink includes global prototypes and class trust. The C×C relation prior is computed locally from those prototypes and is not transmitted. These are logical tensor-payload sizes; they exclude serialization and network-protocol overhead and are not transmission-time measurements.

In the reported implementation, each participating client’s data are traversed to estimate prototypes and dispersions before local optimization for trust calculation and again afterward for upload. For a mini-batch of size *B*, the prototype contrastive computation costs O(BCd), and the class-relation similarity computation costs $O(C^{2}d)$, in addition to feature extraction. The retained semantic SAM branch adds a forward/backward pass on the selected steps despite applying zero parameter perturbation. This is computational overhead without an established sharpness-aware accuracy benefit; the runtime savings from removing the branch have not been measured. The head update-direction upload and non-self direction download are separate from the prototype-and-statistic payload. Each has the size of the classifier head in the reported configuration, approximately 4.52 KiB on UCI HAR and 9.05 KiB on PAMAP2 per participating client and round. Equal backbone size and equal training rounds therefore do not imply equal training cost across methods.

Some archived sensor runs contain elapsed times for the training-and-aggregation loop, excluding evaluation and initialization, but they do not document a controlled hardware benchmark covering all methods and datasets. A CUDA device index alone does not identify the GPU model or concurrent workload. We therefore do not use these partial records to establish a runtime ranking. Standalone inference latency and peak GPU memory were not recorded. The efficiency information here is consequently limited to parameter counts, weight and message sizes, and the operations added by training; a measured comparison of end-to-end training time, inference latency, and peak memory remains outside the reported evaluation.

Class prototypes summarize embeddings rather than raw sensor windows [15], but this distinction does not establish a privacy guarantee. Like shared gradients, these summaries may reveal private information [38]. Section 5 describes the disclosure risks under an honest-but-curious server. Secure aggregation and differential privacy are discussed only as potential future safeguards. Neither was implemented or evaluated, so the reported accuracies, message sizes, and computational costs do not characterize a variant protected by these measures.

## 4. Experiments

We compare FedREPA with ten baselines under a shared Dirichlet label-skew protocol. Clients are simulated label-skew partitions rather than intact subject datasets. Subject-wise client partitions, device-wise partitions, and controlled sensor-placement changes are not evaluated. Sensor-based HAR is the primary application; CIFAR-10/100 provide additional tests of relation-enhanced prototype learning in image classification. Global-accuracy comparisons concern only the evaluated methods and settings on the four datasets. Worst-client comparisons concern only the methods shown in Figure 3 on UCI HAR and CIFAR-10. Neither comparison establishes general robustness to feature shifts or dominance over unevaluated federated HAR methods.

### 4.1. Experimental Setup

#### 4.1.1. Datasets

UCI HAR [1] contains six activities recorded from smartphone accelerometer and gyroscope signals (9 channels, 128 time steps per sample). We use the pre-segmented samples and the official subject-disjoint train/test split shipped with the archive: the 21 training subjects supply the federated training pool and the 9 test subjects form the held-out test set, so no subject appears on both sides. The K=21 clients are obtained by a Dirichlet partition of the training samples [11]. This split evaluates the trained global model on unseen subjects within UCI HAR. However, the federated clients are Dirichlet partitions of the training pool rather than individual subjects, so the experiment does not evaluate subject-wise client heterogeneity.

PAMAP2 [2] provides activity monitoring with multiple inertial measurement units (IMUs); we use 12 protocol activities, a 52-channel input, K=9 clients, and windows of length 128 with stride 64. Because overlapping windows would leak information across a random split, the train/test division is made before windowing: within each subject, every contiguous activity segment is cut temporally; the last 20% of each segment is reserved for testing, and the sliding window is then applied separately inside the two blocks. Train and test windows therefore never share a raw sample. This within-subject temporal holdout does not evaluate transfer to unseen subjects or subject-wise federated clients.

CIFAR-10 and CIFAR-100 [39] use the standard 32×32 splits with K=20 clients. In all four datasets, the label distribution follows Dirichlet α∈{0.1,0.3,0.5,0.8}, where smaller α yields stronger class imbalance and more missing local classes.

#### 4.1.2. Baselines

Within each dataset/α/seed setting, all compared methods use the same benchmark train/test split, preprocessing, and Dirichlet partition of the training pool. The partition is generated from the data, α, and seed independently of the method. The backbone dimensions, client-participation schedule, local epochs, SGD learning rate and weight decay, batch size, and total communication rounds are shared within each dataset. The local SGD momentum is 0 for SCAFFOLD and MoFedSAM to retain their correction rules, and 0.9 for the other implementations. Algorithm-specific losses, update rules, classifier behavior, and auxiliary state are retained, so these controls match the data and training schedule rather than equalizing computational work. All methods are evaluated on the same held-out global test set and evaluation rounds for that dataset, using last-five mean accuracy in the main tables and retrospective best-round accuracy only in Appendix A. For the auxiliary client metrics, the same rule constructs client test subsets from the corresponding training-label distributions. UCI HAR uses the official subject-disjoint split, PAMAP2 uses the temporal split before windowing, and CIFAR-10/100 use their standard train/test splits, as described above. These are comparisons of the implemented methods under this shared protocol; the restricted FedGPS-Core adaptation remains distinct from published FedGPS.

The ten baselines cover the three families identified in Section 2: parameter-space correction (FedAvg [6], FedProx [12] with μ=0.1, SCAFFOLD [13]), representation alignment (MOON [14] with μ=5 and τ=0.5, FedProto [15], FedNH [16]), and sharpness-aware optimization (FedSAM, MoFedSAM [17], FedLESAM [18]). FedGPS-Core completes the set.

FedGPS-Core is our restricted adaptation of FedGPS [19] and is the strongest evaluated baseline. It retains prototype and update-direction cues but omits the published client-side surrogate path. In published FedGPS, clients encode a shared surrogate set drawn from a class-conditional source, such as a Gaussian, and the server aggregates the resulting prototypes; the server does not synthesize the set from uploaded statistics. FedGPS-Core instead applies mean-squared-error prototype alignment to real local features. Omitting the surrogate path and replacing surrogate prototypes are both part of the adaptation and may affect its attainable performance. This baseline is not a reproduction of full FedGPS, and the reported margins apply only to FedGPS-Core under our information-sharing protocol.

FedTGP [21], FedDPA [22], and FedAli-PHAR [23] are discussed in Section 2 but were not evaluated in this study. FedProto and FedNH provide evaluated prototype baselines, but their results cannot substitute for a direct comparison with these three approaches. Accordingly, all numerical rankings and performance margins refer only to the ten evaluated baselines under the protocol specified above. The qualitative distinctions in Section 2 do not establish comparative accuracy, robustness, or efficiency. The absence of these recent approaches remains a limitation, and their relative performance requires direct evaluation.

#### 4.1.3. Implementation

*Architecture*. Sensor datasets use a 1D CNN with GroupNorm [40] (feature dimension d=192). CIFAR-10 uses a SimpleCNN (d=192); CIFAR-100 uses ResNet-18 [41] with GroupNorm (d=512).

*Training*. Local training uses SGD with weight decay $5\times 10^{-4}$ and batch size 64. The local optimizer uses momentum 0.9 except for SCAFFOLD and MoFedSAM, which use momentum 0 with their algorithm-specific correction rules. The learning rate is 0.01 on all datasets except PAMAP2, where it is 0.005. UCI HAR, PAMAP2, and CIFAR-10 use 100 communication rounds with 2, 2, and 3 local epochs, respectively. CIFAR-100 uses 200 rounds with 3 local epochs. Each round samples 50% of clients (67% on PAMAP2).

*FedREPA hyperparameters*. We use $\lambda_{1}=0.3$, $\lambda_{2}=1.0$, κ=1.0, $T_{r}=1.0$, JS for $L_{\mathrm{rel}}$, and a fixed contrastive temperature τ=0.1. The relation loss starts after a warm-up of $T_{\mathrm{rel}}=10$ rounds on UCI HAR, PAMAP2, and CIFAR-100, and 5 rounds on CIFAR-10; the prototype contrastive loss is active from the first round. A separate warm-up for $L_{\mathrm{pcon}}$ is not used. As described in Section 3, the random initialization is mixed with the uploaded class mean at every round, with μ=0.5, rather than being replaced after round t=1. The within-round relation cache uses $\beta_{\mathrm{rel}}=0.5$, and the missing-class mask requires $n_{\min}=2$ samples. Trust uses $\tau_{d}=\tau_{v}=1.0$, $n_{0}=50$, $q_{\min}=0.1$, EMA 0.9, and aggregation exponent η=1.0 with $\tau_{\mathrm{dev}}=0.5$; prototypes are updated with EMA coefficient 0.5. The reported semantic SAM setting selects only the output classifier head, not the feature projection. The semantic backward pass is scheduled every second local step after $T_{\mathrm{sam}}=50$ rounds for the 100-round datasets and 100 rounds for CIFAR-100. Its configured class-wise radius is ρ∈[0.005,0.02], and its non-self direction weight is 0.15. These values document the executed implementation; the actual semantic perturbation is zero throughout the reported head-only runs. Neither the configured radius nor the non-self weight implies an effective sharpness-aware update. The full local loss is differentiated at unperturbed weights, and no accuracy improvement is attributed to semantic SAM. Results are mean ± SD over seeds {2024,2025,2026}. The main tables use κ=1.0 and $T_{r}=1.0$. A separate UCI HAR sensitivity sweep varies κ∈{0.5,1,2} at $T_{r}=1$ and $T_{r}\in \left\{0.5,1,2\right\}$ at κ=1, with its own κ=1, $T_{r}=1$ anchor. This sweep is separate from the main tables and does not establish corresponding changes on PAMAP2 or CIFAR.

The main settings were fixed using the disjoint validation procedure described below; they are not claimed to be global optima. The values κ=1 and $T_{r}=1$ define the central point for the one-factor UCI HAR sweep. At this temperature, the relation distribution remains relatively flat and induces moderate negative weights (approximately 1.7–2.3 in the reported UCI HAR example), rather than an extreme emphasis on a few classes. The tested alternatives at 0.5 and 2 shift mean accuracy by less than one percentage point in that sweep, with mixed seed-level directions, so the default is retained as a moderate reference setting rather than a demonstrated winner. The relation-loss warm-up delays matching until the global prototypes have begun to incorporate client information, while $L_{\mathrm{pcon}}$ remains active from the first round to provide cross-client negatives under sparse local class coverage. The threshold $n_{\min}=2$ excludes singleton class observations from relation matching. The loss weights and remaining fixed coefficients were selected under the disjoint validation procedure described below and are stated for reproducibility; no individual sweep establishes their separate optimum.

#### 4.1.4. Metrics

Accuracy and recall levels are expressed as percentages (%); absolute differences are expressed in percentage points (pp), whereas explicitly identified relative gains are percentages. The main tables report global test accuracy (%) averaged over the last five evaluation points of each fixed training schedule, without selecting a round by its test accuracy. This is an end-of-schedule summary, not the accuracy of a single deployed checkpoint. Appendix A reports the maximum test accuracy attained by the same runs. This retrospective best-round score is optimistic and is not a test-independent checkpoint-selection procedure. FedREPA has the highest reported mean among the eleven evaluated methods under both conventions, but the complete method ordering is unchanged in only 15 of the 16 dataset/α columns. On CIFAR-100 at α=0.8, last-five scoring places FedNH 0.01 points above FedSAM, whereas best-round scoring places FedSAM 0.86 points above FedNH. This reversal illustrates the scoring dependence of some baseline comparisons. The auxiliary metrics in Section 4.3 retain last-five scoring; no best-round counterpart is reported for them.

Two auxiliary metrics are used for analysis only. *Selected-class recall* is the mean per-class recall over the k=max(1,⌊C/5⌋) classes with the smallest test-set counts, which is one class on UCI HAR (C=6) and two on CIFAR-10 (C=10). On the official UCI HAR test set, the six activity counts are 496, 471, 420, 491, 532, and 537, so the single selected class is Walking Downstairs. The official CIFAR-10 test set has 1000 images in every class, so the count vector is constant and does not rank classes. The evaluation takes the first k=2 indices of torch.argsort on that vector. In PyTorch 2.7.1, that call returns indices 0 and 1, airplane and automobile in the CIFAR-10 order used by the loader. The CIFAR-10 value is the mean recall of those two classes. It is not a frequency tail and is not evidence about rare classes. *Worst-client accuracy* is the minimum over all *K* clients, participating or not, of the accuracy of the current global model on a local test subset whose label distribution mirrors that client’s training mix.

#### 4.1.5. Statistical Reporting

Each main-comparison cell summarizes three independently seeded runs (2024, 2025, and 2026). A run is first scored by its mean over the last five evaluation points; the reported mean and standard deviation (SD) are then computed across the three run scores. The reported SD is the descriptive root-mean-square deviation with divisor three (ddof = 0), matching the table-generation and plotting code. The five points from one training trajectory are not independent repeats and do not increase the sample size from three to fifteen. The standard deviation describes between-run dispersion; it is neither a standard error nor a confidence interval for a method difference. With only three runs, both the mean and the standard deviation are sensitive to individual runs. Numerical rank, small gaps, or overlap of standard-deviation bands alone do not establish statistical superiority, inferiority, or equivalence. The main comparison, including the paired margins against FedGPS-Core, is descriptive only and makes no confirmatory inferential claims. Section 4.2 explains the dependence created by seed reuse across α.

#### 4.1.6. Model Selection

Hyperparameters were selected once per dataset using a validation split disjoint from the test set and were not subsequently retuned. On UCI HAR, the split holds out complete training subjects (ratio 0.2, a fixed selection seed distinct from the three evaluation seeds); on PAMAP2, it is a second temporal cut inside the training block of each activity segment, so the test block plays no role. Candidates were ranked by the mean macro-F1 of the last five validation evaluations across the three seeds. The selected configuration was then evaluated under the stated test protocol. Neither selected-class recall nor worst-client accuracy was used for model selection.

### 4.2. Main Results

Table 3, Table 4, Table 5 and Table 6 report the 11-method comparison. Within this evaluated set, FedREPA has the highest observed mean global accuracy in all 16 dataset/α settings under Dirichlet label skew. Averaged over the four α values, the reported accuracies are 90.96% on UCI HAR, 98.27% on PAMAP2, 76.57% on CIFAR-10, and 58.87% on CIFAR-100. The mean margins over the strongest evaluated baseline, FedGPS-Core, are approximately +0.99, +1.00, +1.02, and +1.04 percentage points, respectively. These are consistent directions of the observed differences in global accuracy, not evidence of uniform improvement in every metric or statistically established superiority in every cell. The comparison is with restricted FedGPS-Core, not published FedGPS, and does not include FedTGP, FedDPA, or FedAli-PHAR. The mean relative accuracy gain over FedAvg is 14.67% across the four datasets; this percentage is distinct from the absolute percentage-point margin over FedGPS-Core. We use the stronger FedGPS-Core baseline as the primary reference to avoid overstating the gain by comparison with FedAvg alone.

**Figure 2:**
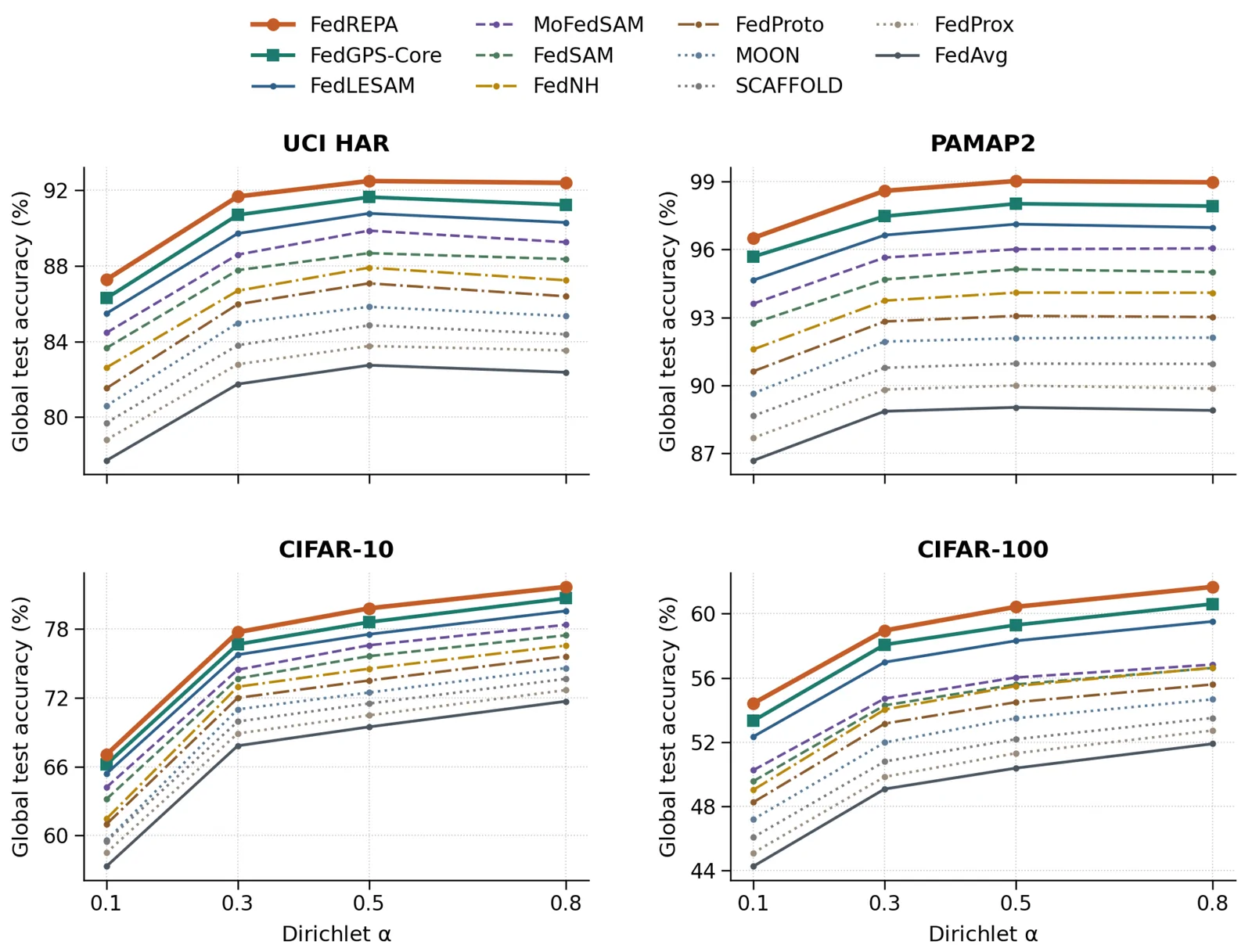
Mean global test accuracy (%) versus Dirichlet α on four datasets, using the mean of the last five evaluation points per run and then averaging three seeds, as in Table 3, Table 4, Table 5 and Table 6. FedREPA (orange) has a higher reported mean than FedGPS-Core (teal) at each tested level; this is a descriptive ranking, not a significance statement. Lower α is stronger label skew, not a perturbation-robustness test.

Figure 2 summarizes the mean global-accuracy results across α. FedREPA remains numerically above FedGPS-Core at the tested levels, including α=0.1. The approximately 10-point margin over FedAvg does not systematically widen at the most skewed setting, so these results do not establish a preferential benefit under more severe label skew. The lower absolute accuracy on CIFAR-100 also shows that the stable top ranking does not imply comparable task difficulty or absolute performance across datasets. The mechanism responsible for the differences is a separate question from this observed ranking.

For a descriptive paired comparison, each FedREPA run is matched with the FedGPS-Core run using the same dataset, α, and seed. All 4×4×3=48 observed accuracy differences are positive, ranging from +0.57 to +1.52 percentage points (mean +1.01, standard deviation 0.19). The cross-seed spread of up to 7.2 points on UCI HAR is the largest gap between the three seeds of one method at α=0.1, not the uncertainty of the paired difference. The per-dataset means of the paired margins are +0.99, +1.00, +1.02, and +1.04 on UCI HAR, PAMAP2, CIFAR-10, and CIFAR-100. These figures describe this grid.

Across values of α, runs that share a seed reuse the initialization and the client-sampling sequence. The clients in round *r* are drawn from a generator seeded by seed+r, which does not include α. The Dirichlet partition is redrawn from the same seed with concentration α, so it is not a copy, but the shared initialization and client sampling mean that the 48 pairs are not independent replicates.

Accordingly, the paired comparison is descriptive only and makes no confirmatory inferential claims. No hierarchical or mixed-effects model accounting for this seed reuse was fitted. Neither the positive sign pattern nor the mean margin is used to establish statistical superiority. The 0.19-point standard deviation describes dispersion among the observed margins; it is not a standard error or a confidence interval for an overall method effect. No pooled sign test or i.i.d. confidence interval is used for this comparison. These limitations also apply to the per-dataset summaries.

### 4.3. Selected-Class Recall and Worst-Client Accuracy

**Figure 3:**
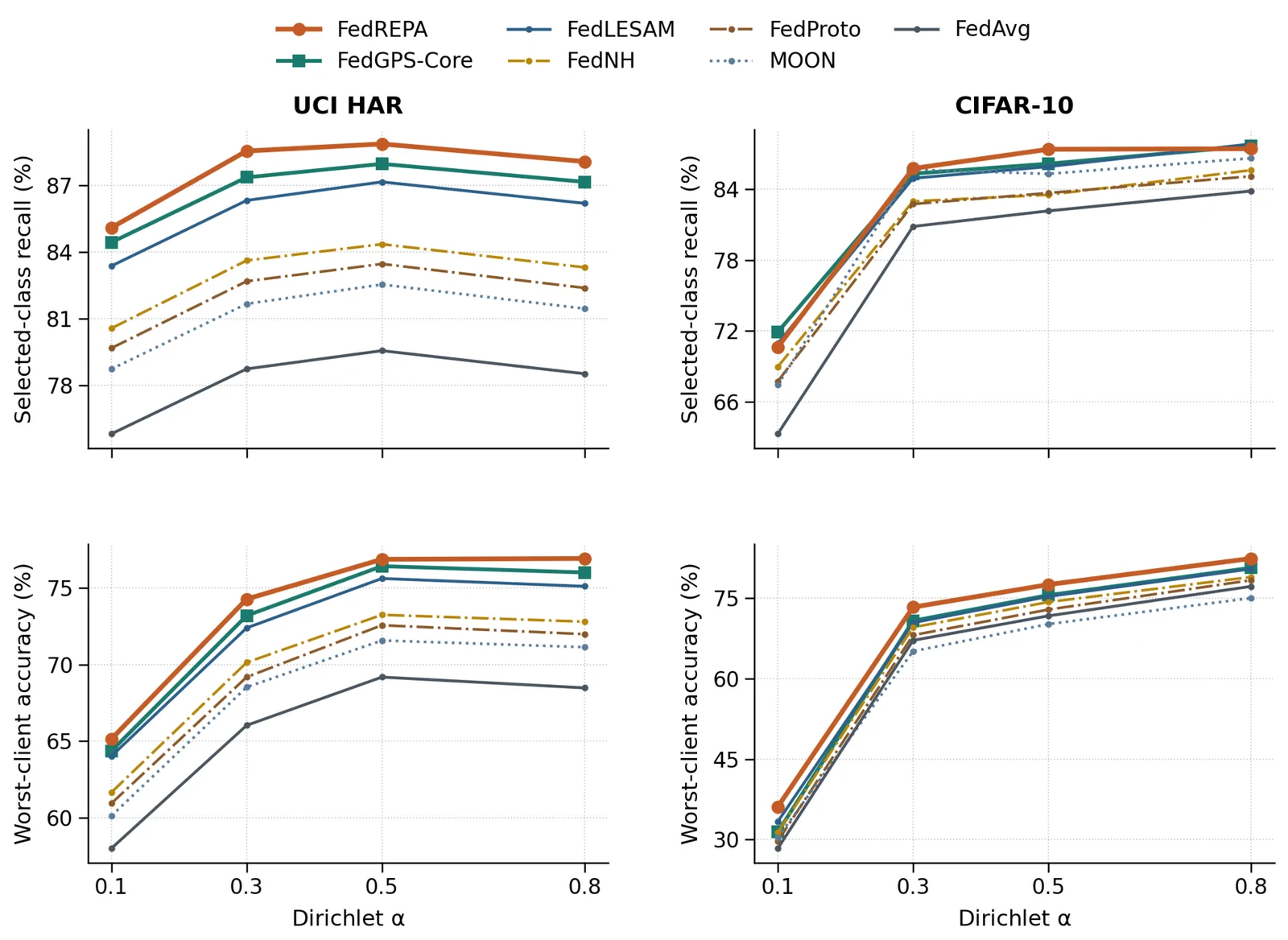
Selected-class recall (%, **top**) and worst-client accuracy (%, **bottom**) versus Dirichlet α, averaged over the last five evaluation points and three seeds. FedREPA (orange) has the highest observed worst-client accuracy among the methods shown on UCI HAR and CIFAR-10. The top-panel ranking is not uniform: FedGPS-Core is ahead on CIFAR-10 at α=0.1 and α=0.8 by 1.3 and 0.2 percentage points. The UCI HAR top panel is the recall of Walking Downstairs, the least frequent test class; the CIFAR-10 top panel is the mean recall of airplane and automobile, selected by sorting equal test-class counts, and is not a rare-class comparison. These panels do not establish dominance across all classes, metrics, or datasets.

The auxiliary results in Figure 3 are metric-specific. FedREPA has the highest observed worst-client accuracy at each tested Dirichlet level on UCI HAR and CIFAR-10 among the methods shown. At α=0.1, the reported values are 65.1% versus 58.0% for FedAvg on UCI HAR and 36.1% versus 31.4% for FedGPS-Core on CIFAR-10. These results concern the constructed client test subsets on those two datasets; corresponding results are not reported for PAMAP2 or CIFAR-100. The selected-class recall ranking differs: FedGPS-Core is ahead of FedREPA on CIFAR-10 at α=0.1 and α=0.8 by 1.3 and 0.2 percentage points. These exceptions preclude a claim of improvement in every auxiliary metric, while three seeds provide limited support for interpreting small differences in either direction. On UCI HAR, the selected-class metric is the recall of Walking Downstairs, the least frequent test class. On balanced CIFAR-10, it is the mean recall of airplane and automobile, selected by the count-sorting rule, not a rare-class metric. Neither panel establishes improvement for every class or general fairness across users.

### 4.4. Ablation Studies

**Figure 4:**
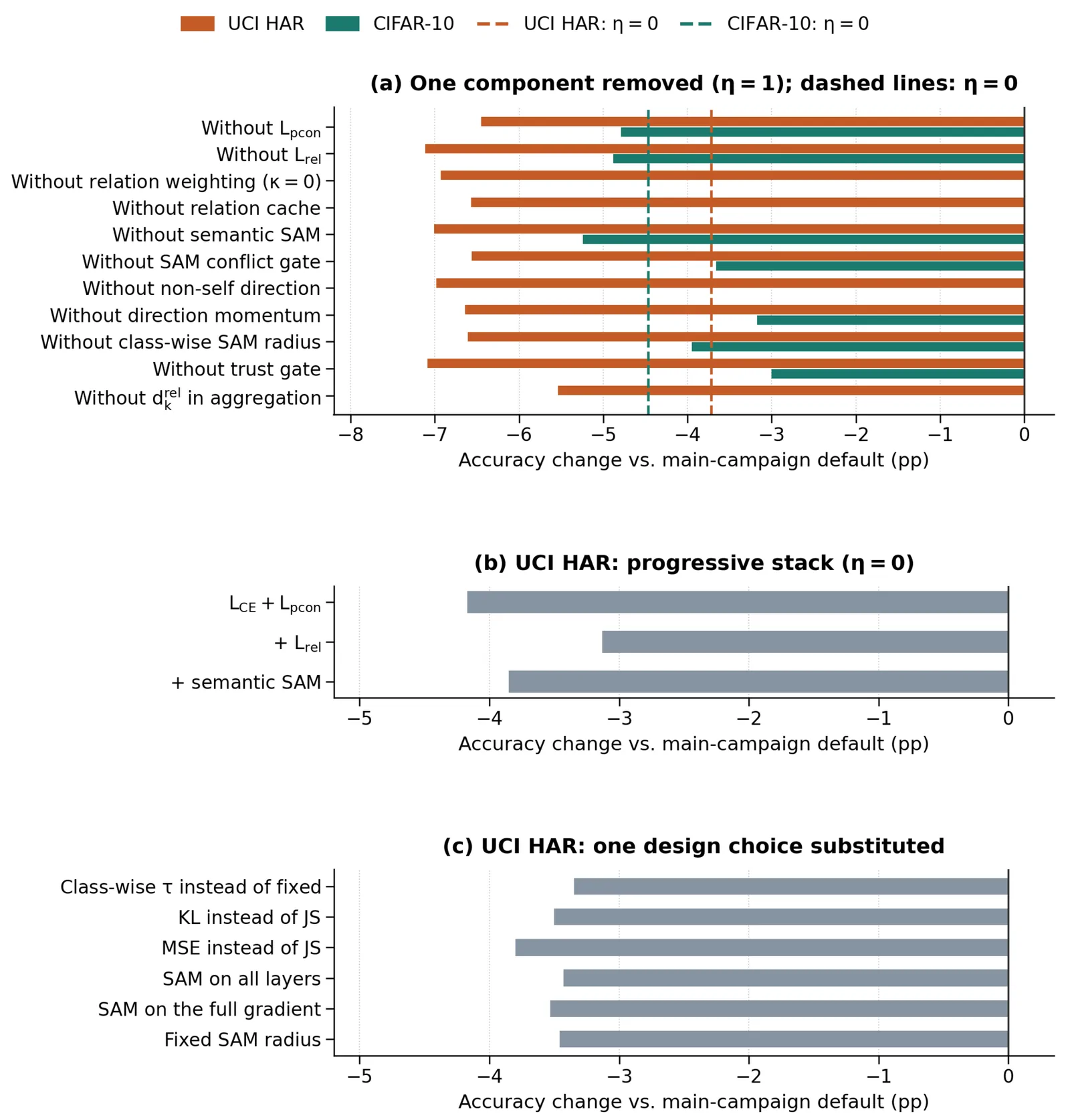
Ablations as accuracy changes in percentage points (pp) from the main-campaign default. (**a**) Removing one component with η=1; the orange and teal dashed lines mark the η=0 fallback for UCI HAR and CIFAR-10, respectively. Every UCI HAR removal falls below its fallback accuracy, whereas removing the trust gate on CIFAR-10 gives 73.57%, above its fallback of 72.10%. (**b**) Progressive stacking under FedAvg aggregation on UCI HAR. (**c**) Substituting one design choice on UCI HAR. Missing CIFAR-10 bars in (**a**) correspond to unreported entries in Table 7. The zero line is the main-campaign default, not a same-campaign rerun. No confidence intervals, significance thresholds, or equivalence bands are shown; all reference-row gaps remain exploratory. SAM-related bars do not measure the benefit of an effective perturbation in the default head-only configuration.

The ablation runs use the protocol, seeds, and α grid of Table 3, Table 4, Table 5 and Table 6. They examine component removals, progressive loss combinations, and substitutions of design choices. Table 7 and Figure 4 group the 21 variants into three blocks that must be read separately, because the blocks differ in the aggregation rule: (A) one component removed while trust-weighted aggregation stays on (η=1), (B) losses stacked progressively under plain FedAvg aggregation (η=0), and (C) one design choice substituted by an alternative of the same kind.

The original ablation grid does not contain a rerun of the unmodified default, so its reference row comes from the main-comparison campaign. Gaps from that row mix the ablation with any between-campaign offset and remain exploratory. Within-block comparisons avoid that particular offset but are still based on only three seeds. The 1.02-point observed repeat-run spread, calculated below from two executions of the same configuration, is a descriptive repeatability check, not a confidence interval, a significance cutoff, or an equivalence margin. Differences smaller than this spread are particularly difficult to interpret; differences exceeding it are not automatically statistically significant. The spread also does not bound the offset from the main campaign, so even the larger reference-row gaps are not calibrated component effects.

Within block A, removing either core loss gives large observed gaps on both datasets: $L_{\mathrm{pcon}}$ is 6.45 and 4.79 points below the reference, and $L_{\mathrm{rel}}$ is 7.11 and 4.88 points below it, on UCI HAR and CIFAR-10. Because the default row is from another campaign, these are cross-campaign associations, not calibrated component effects. Setting κ=0 leaves the prior and $L_{\mathrm{rel}}$ active but changes both the relative weights and their total scale: for uniform $\pi_{g}$, κ=1 gives $2{\tilde{q}}_{c}$ and κ=0 gives ${\tilde{q}}_{c}$. The same-campaign ten-seed comparison supports the tested κ=1 weighting configuration over κ=0, and the selected class-pair error rates move in the same direction. However, neither contrast isolates the benefit of a non-uniform relation prior, because no control kept κ=1 while replacing $\pi_{g}$ with the uniform distribution. Thus, the evidence supports the tested combination, not a separate causal attribution to relation non-uniformity alone. The original table’s κ=0 row keeps trust-weighted aggregation (η=1); a separate UCI HAR campaign also tested this contrast at η=0 and is reported as a distinct analysis.

Block A does not establish independent or additive contributions from the optional modules. Every single-removal value in that column is below the η=0 row (87.24), but this comparison is against the main-campaign reference and is exploratory. The CIFAR-10 pattern differs: removing the trust gate gives 73.57, above the η=0 entry 72.10. In the separate ten-seed UCI HAR campaign, the four-α mean changes by only about 0.08 points between the full configuration at η=1 and η=0. Thus, these results do not establish a consistent accuracy benefit from trust-weighted aggregation, nor that each optional aggregation component improves performance on every dataset.

Block B compares stacked losses under FedAvg aggregation. Its three variants span 86.79–87.83, about one percentage point. As a descriptive repeatability check, two grid entries share an identical configuration and identical seeds (η=0 with all other modules enabled, reached once as a removal and once as the top of the stack). Their largest absolute per-run difference is 3.11 points (α=0.3, seed 2026). The differences of the three-seed means are +0.849, +0.937, −2.249, and −0.061 points at α=0.1, 0.3, 0.5, and 0.8. Their mean absolute value is (0.849+0.937+2.249+0.061)/4=1.024, rounded to 1.02 points. The two four-α means differ by 0.13 points (87.24 and 87.11). These repeated executions illustrate variation in the reported grid; they do not estimate a general significance threshold. We therefore do not interpret the small block-B differences as reliable improvements or decrements, including a claim that semantic SAM helps or hurts.

In the progressive stack, adding $L_{\mathrm{rel}}$ changes the reported mean from 86.79 to 87.83 (+1.04 points), while adding the reported head-only semantic SAM setting changes it from 87.83 to 87.11 (−0.72 points). These are three-seed descriptive differences, not reliable estimates that the relation loss improves or semantic SAM harms performance. Because the reported head-only perturbation is zero, the latter contrast is between execution branches rather than between an effective semantic perturbation and its removal. The extra loss evaluation and relation-cache update also differ, and their effects were not isolated. Neither this difference nor the larger cross-campaign gap for removing semantic SAM establishes its benefit or necessity.

Block C compares substitutions among loss and perturbation designs. Its six variants fall within 0.45 points of one another (87.16–87.61 on UCI HAR), but this narrow numerical range in three-seed means does not rank the alternatives or establish equivalence. Crucially, in the reported head-only SAM setting, the semantic gradient on the selected classifier is zero, so the perturbation is zero; its ablation cannot be interpreted as evidence that a nonzero semantic perturbation improves accuracy. The “SAM on all layers” and “SAM on the full gradient” variants change the parameters or loss used to construct the perturbation and are different operations from the default. Removing the conflict gate can introduce a non-self perturbation when a nonzero direction is available. In contrast, removing the non-self direction while retaining the enabled gate leaves the zero default perturbation unchanged; changing its momentum or scaling a zero direction by a different radius also does not create a perturbation. Accuracy differences in these rows therefore do not validate the default semantic SAM mechanism. Their causes are not isolated by the reported comparisons.

The distinct roles of relation consistency and prototype contrastive learning describe what the objectives optimize; they do not quantify separate contributions to the total accuracy gain. The original ablation table provides exploratory associations between configuration changes and accuracy, rather than a calibrated attribution of the total gain to individual modules. Its cross-campaign reference, three-seed evaluation, and coupled aggregation rule limit component-level conclusions. More independent seeds and a same-campaign rerun of the default would be needed for stronger comparisons. Small differences remain unresolved, and neither a larger numerical gap nor a gap exceeding the observed repeat-run spread is treated as a significance result.

### 4.5. Convergence Behavior

**Figure 5:**
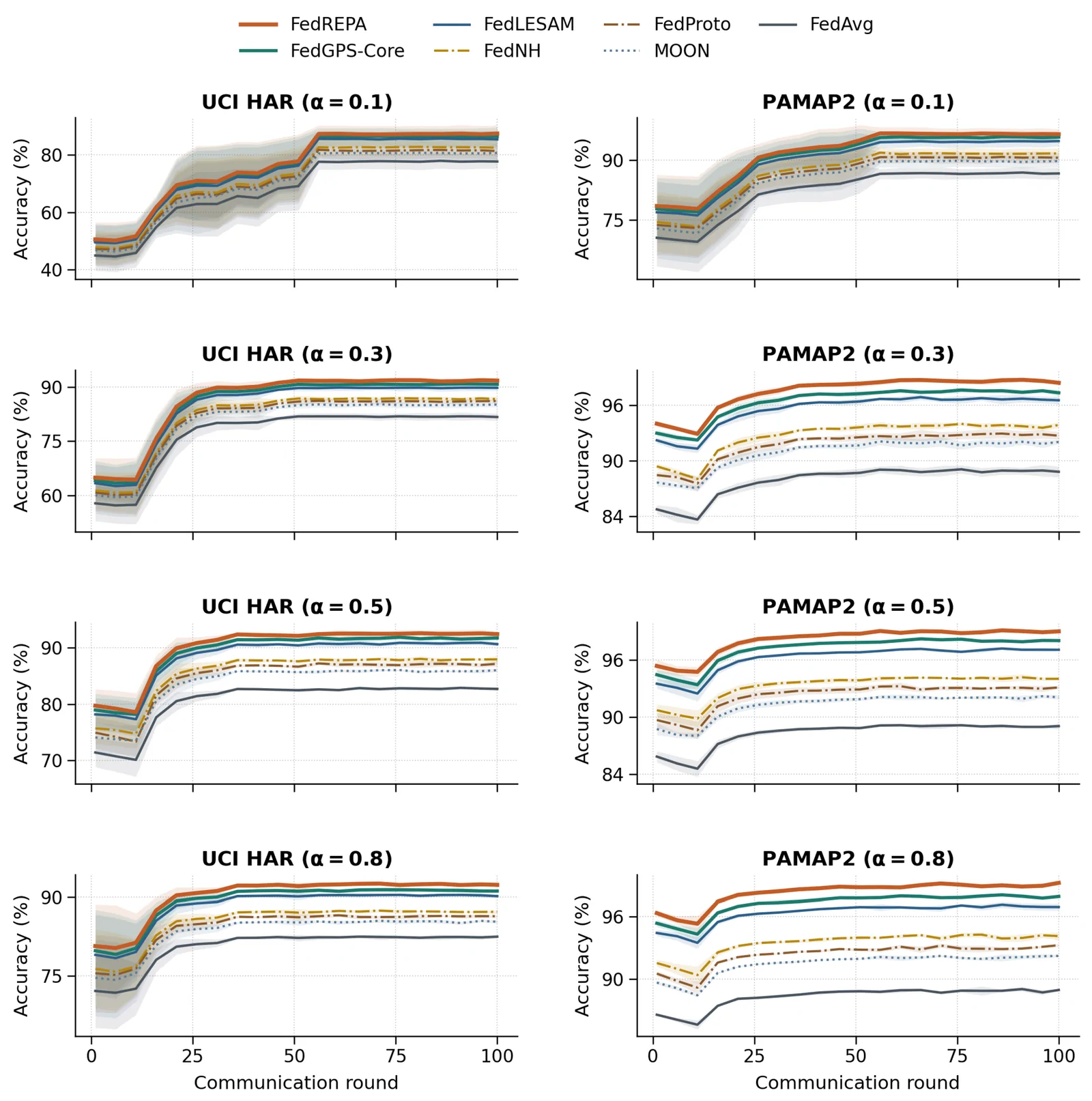
Convergence on UCI HAR and PAMAP2 for α∈{0.1,0.3,0.5,0.8}. Rows correspond to the four increasing α values and columns to the two datasets. Orange: FedREPA; teal: FedGPS-Core. The vertical axis is accuracy (%), and the horizontal axis is communication round, not elapsed time. Shaded regions denote one SD over three seeds (ddof = 0), not confidence intervals.

**Figure 6:**
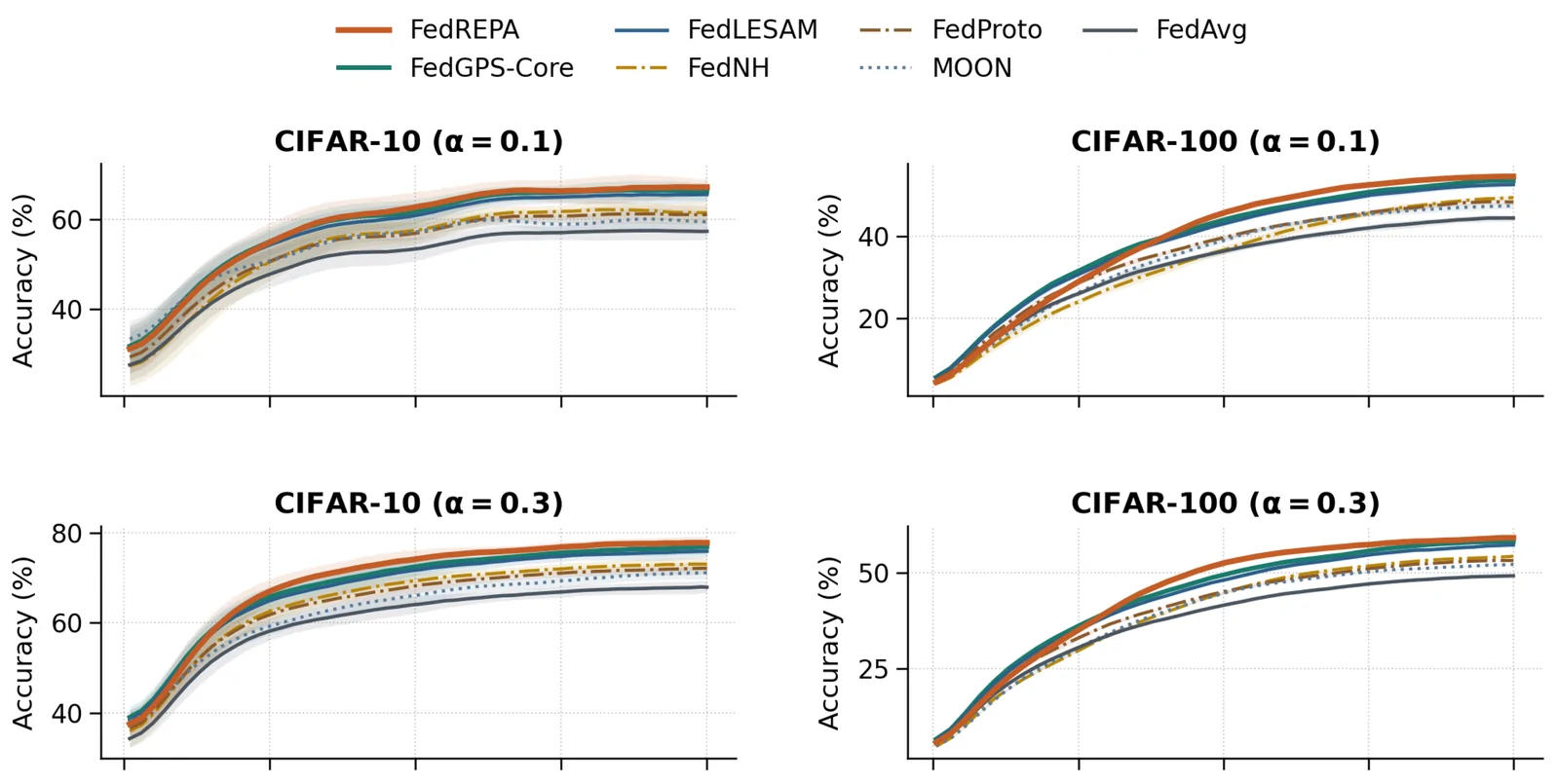

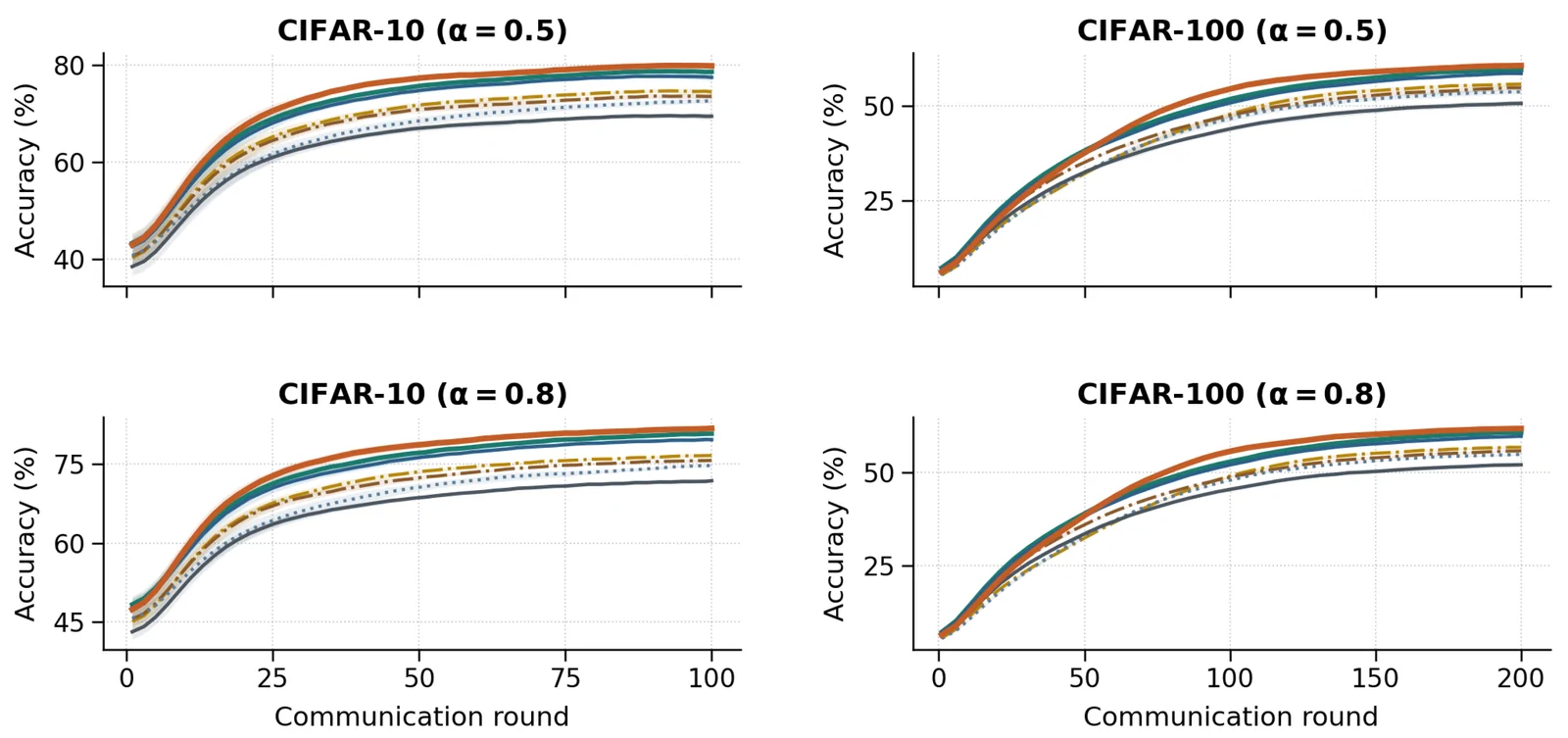
Convergence on CIFAR-10 and CIFAR-100 for α∈{0.1,0.3,0.5,0.8}. Rows correspond to the four increasing α values and columns to the two datasets. Orange: FedREPA; teal: FedGPS-Core. The vertical axis is accuracy (%), and the horizontal axis is communication round, not elapsed time. Shaded regions denote one SD over three seeds (ddof = 0), not confidence intervals.

Figure 5 and Figure 6 summarize global test accuracy versus communication round at the four Dirichlet levels α∈{0.1,0.3,0.5,0.8} (mean ± SD over three seeds). They describe training trajectories for the selected baselines, not independently tested superiority at every round. The sensor trajectories generally level off earlier than the image trajectories, although UCI HAR at α=0.1 has an additional rise around rounds 50–60. CIFAR-10/100 rise more gradually, and CIFAR-100 uses a 200-round schedule. At α=0.1, wide, overlapping cross-seed bands limit the interpretation of small round-wise differences. The descriptive paired end-of-schedule comparison is reported in Section 4.2. Neither these bands nor the communication-round axis establish perturbation robustness or an advantage in wall-clock training time.

These curves describe empirical training behavior rather than a formal convergence guarantee. FedREPA modifies the local objective and, when η>0, the aggregation weights. FedAvg convergence guarantees under bounded heterogeneity therefore do not transfer directly; analysis of the relation-weighted objective remains future work.

## 5. Discussion

The most consistent comparative pattern is the higher observed mean global accuracy of the complete FedREPA configuration across the tested dataset/α settings. This pattern does not extend to every auxiliary metric: the CIFAR-10 selected-class recall has two exceptions, and worst-client results are available only for UCI HAR and CIFAR-10. These findings support a bounded description of the reported configuration, not universal superiority or an isolated causal effect of the relation prior. Prototype proximity motivates class-dependent negative weighting, but proximity is not itself a measured confusion rate. In addition, the κ=0 contrast changes the overall negative-weight scale as well as the relative weights, so it does not by itself establish that non-uniform relation information explains the global-accuracy ranking.

The main tables report global test accuracy, while Figure 3 reports selected-class recall and worst-client accuracy. The separate paired campaign measures the selected class-pair error rates. Prototype cosine proximity is a plausible proxy for confusability, but it is not the same quantity as the confusion rate. A separate UCI HAR campaign compares κ=1 with κ=0 at η=1, for α∈{0.1,0.3,0.5,0.8} and seeds {2024,…,2033}. It does not replace the cells in Table 3, Table 4, Table 5 and Table 6. Last-five test accuracy averages 87.46%, 91.56%, 92.36%, and 92.48% for κ=1 and 80.30%, 83.40%, 84.13%, and 87.67% for κ=0. Averaging the four α values within each seed, the accuracy drop at κ=0 has a mean of 7.09 percentage points (range 6.08–8.07), and accuracy decreases for all ten seeds. Confusion counts come from the same evaluations. The mean seed-level rate of misclassifying Sitting as Standing rises from 1.10% to 4.64% at κ=0, Standing predicted as Sitting from 1.15% to 5.36%, Walking predicted as Walking Upstairs from 1.13% to 5.27%, and Walking Upstairs predicted as Walking from 1.17% to 4.97%. Each directed error rate is higher at κ=0 for all ten seeds. Across α, a shared seed reuses initialization and the client-sampling sequence, so the forty α–seed pairs are not independent replicates. The seed is the independent unit. A two-sided sign-flip test on the ten seed-level reductions gives $p=2/2^{10}=0.001953$, reported as p=0.002, for each of the four rates. The four seed-level reductions span 3.41–3.68, 3.97–4.35, 3.97–4.31, and 3.67–3.97 percentage points. Applying Holm’s correction across the four tests gives 0.0078, which remains below 0.05. That test concerns these class-pair error rates on UCI HAR. It is not a pooled test of the forty pairs; it does not cover PAMAP2 or CIFAR, and it does not replace the descriptive 48-pair comparison with FedGPS-Core. The κ=0 row of the ablation table remains a comparison against the main-campaign reference. The paired UCI HAR campaign above is the same-campaign estimate of that contrast (Table 8).

Worst-client accuracy offers a complementary view of the evaluated label-skew partitions. At α=0.1, its observed margins are 7.1 percentage points over FedAvg on UCI HAR and 4.7 points over FedGPS-Core on CIFAR-10. These examples use different reference baselines and should not be combined into a common effect size. They concern the minimum accuracy over constructed client test subsets, not a clinical endpoint or demonstrated robustness to atypical movements, subject identity, or device shift. Providing cross-client negative prototypes is a possible explanation, but these aggregate comparisons do not isolate that mechanism.

Two findings temper the method’s presentation. The first concerns the strength of the prior. At $T_{r}=1$, the rows of $\pi_{g}$ span 0.14–0.26 around the uniform value 1/(C−1)=0.2, so the induced weights span roughly 1.7–2.3: the mechanism is a moderate reweighting, not an extreme hard-negative emphasis. This leaves open whether the observed gain reflects relation information or the use of global prototypes as negatives. The κ=0 comparison retains the prototypes and $L_{\mathrm{rel}}$, but it changes both the relative weights and their overall scale. Its original ablation-table gap is cross-campaign, and the separate ten-seed comparison also changes that scale. Neither contrast isolates the contribution of a non-uniform prior.

A uniform-prior control would retain κ=1, global prototypes as anchors, trust weighting, the relation-consistency objective, and the training protocol, but replace $\pi_{g}\left(y,c\right)$ only in (5) by 1/(C−1) for c≠y. This gives $w_{c}\left(y\right)=2{\tilde{q}}_{c}$ for each negative class and removes the class-pair dependence of the relation factor while retaining the trust gate. This uniform-prior control was not run in the reported evaluation. Consequently, the observed κ=1 versus κ=0 differences do not establish that non-uniform relation information, rather than the change in overall negative-weight scale, causes the accuracy gain. Evaluating this control with matched seeds and configurations remains future work.

The completed UCI HAR sensitivity sweep uses its own κ=1, $T_{r}=1$ anchor (four-α mean 90.92%) and varies one coordinate at a time: κ∈{0.5,2} at $T_{r}=1$, and $T_{r}\in \left\{0.5,2\right\}$ at κ=1. Across the ten seeds, the mean changes are −0.39, +0.32, −0.21, and +0.45 percentage points, respectively; the seed-level ranges are −0.96 to +0.12, −0.61 to +0.93, −1.24 to +1.02, and −0.42 to +1.08. The positive-seed counts are 3/10, 7/10, 3/10, and 8/10. These small, mixed-direction differences do not provide a reliable ranking or establish equivalence. The sweep is limited to UCI HAR and does not establish that other settings perform better on PAMAP2 or CIFAR. Extending the sensitivity analysis beyond UCI HAR is future work (Table 9).

The second issue is the limited evidence for contributions from optional modules. The ablations in the original UCI HAR table show every removal falling below the η=0 row (87.24). This pattern does not hold across all datasets. On CIFAR-10, removing the trust gate gives 73.57, which is above the η=0 entry 72.10. In the separate UCI HAR campaign, the full model differs by about 0.08 points between η=1 (90.97%) and η=0 (90.89%). This numerical ordering does not establish that the modules must be used jointly. In particular, the zero semantic perturbation cannot explain an improvement through sharpness-aware optimization.

Several limitations remain. The controlled heterogeneity factor in our experiments is the client label distribution, varied through Dirichlet partitioning. Subject-wise client partitions, cross-device evaluation, and controlled sensor-placement changes were not included. Natural differences already present in the benchmark recordings do not substitute for controlled evaluations of these factors. Feature-level differences are a distinct challenge addressed by on-device signal alignment methods [33]. Our comparative conclusions are therefore restricted to the evaluated benchmark splits and simulated Dirichlet label-skew settings. The sensor benchmarks have few classes (C=6 and C=12), so the relation prior is a small matrix, and its benefit on activity taxonomies with dozens of fine-grained classes is untested.

### Generalizability and Clinical Applicability

External validity is limited by the use of public benchmarks rather than an external institutional or clinical cohort. The UCI HAR subject-disjoint test split evaluates unseen subjects within that benchmark, not transfer to a new institution. PAMAP2 uses within-subject temporal holdouts. The Dirichlet clients are simulated label-skew partitions, not hospitals or care providers, and no cross-institution or prospective clinical evaluation was conducted.

Acquisition protocols are another untested source of shift. Sensor placement, orientation, sampling frequency, calibration, device hardware, preprocessing, and activity annotation were not varied systematically. CIFAR-10/100 provide natural-image classification tests, not medical-imaging validation; those results do not establish transfer across imaging protocols or clinical imaging sites.

Clinical applicability remains a possible future use, not an established outcome. Performance in clinically defined populations, including people with impaired mobility, was not separately evaluated, and no diagnostic, treatment, or patient-outcome endpoint was assessed. Window-level benchmark accuracy and the constructed worst-client metric do not establish reliable continuous monitoring in clinical practice. Robustness to missing or noisy sensor streams, detection of unknown activities, real-time operation, and integration into clinical workflows were not evaluated. Assessing a healthcare application would require external and prospective validation, target-population error analysis, device-level runtime testing, workflow assessment, and privacy safeguards for the shared class statistics.

Three additional evaluation limits apply. First, the comparison excludes the 2024–2026 prototype methods reviewed in Section 2, namely FedTGP, FedDPA, and FedAli-PHAR. The ranking therefore concerns only the ten evaluated baselines and does not establish state-of-the-art performance. The qualitative discussion of these recent approaches does not substitute for a direct experimental comparison. Second, FedGPS-Core omits the client-side surrogate path of published FedGPS and aligns prototypes on real local features; its reported margin is not a margin over full FedGPS. Third, the main results average the last five evaluation points of fixed schedules without early stopping. They do not assess methods under longer or adaptively terminated schedules.

The main evaluation uses only three independent seeds per dataset/α cell. Its means and standard deviations are limited summaries of training variability under the fixed benchmark protocol, not precise estimates of comparative uncertainty or generalization to new populations. Pairing methods by seed is useful descriptively but does not resolve the small number of repeats or the dependence created by reusing seeds across α. The paired comparison is descriptive only; no hierarchical or mixed-effects model accounting for seed reuse was fitted, and no confirmatory inference is made for the main comparison. Small ablation and auxiliary-metric gaps are not interpreted as established benefits, harms, or equivalence. The separate ten-seed UCI HAR analyses address their specified contrasts only; they do not increase the number of independent repeats in the four-dataset main comparison. The original ablation reference remains cross-campaign, and its 1.02-point repeat-run spread is not a significance threshold. More independent runs and an analysis accounting for the repeated-seed design are needed for stronger inferential conclusions.

Keeping raw sensor data local does not prevent disclosure through the shared statistics. The threat model is an honest-but-curious server that stores every uploaded object, together with clients that do not collude. Beyond the parameter update, a FedREPA client transmits its prototypes $P_{k}$, class counts $n_{k,c}$, dispersions ${\mathrm{Var}}_{k,c}$, and trust scores $q_{k,c}$. The class counts $n_{k,c}$ directly reveal the client’s activity histogram. An honest-but-curious server therefore learns how many windows of lying, sitting, or stair climbing each participant contributed and how that composition changes across rounds. In a potential healthcare or eldercare application, this histogram may itself be sensitive, despite the absence of raw-signal uploads. Prototypes and dispersions also expose class-conditional feature summaries [15,38]. FedREPA therefore adds a semantic information channel beyond model updates. Trust scores are also visible to the server and summarize prototype agreement, dispersion, and sample support; their use as reliability weights does not conceal these inputs. This discussion identifies exposure through the specified messages; it is not an empirical measurement of reconstruction or inference risk.

Secure aggregation and differential privacy are possible directions for future work. Neither was implemented or experimentally evaluated in the reported runs. A secure-aggregation extension would require a specified protocol for computing the trust-weighted model and prototype aggregates without exposing the individual client inputs currently received by the server; this protocol and its protection have not been established here. A differential-privacy extension would require a defined privacy mechanism and analysis of the information released through model updates and all auxiliary statistics over repeated rounds. No privacy budget or end-to-end privacy guarantee is established in this study. The effects of either extension on accuracy, aggregation behavior, communication, and runtime remain unmeasured; no claim is made that the utility cost would be small. These possibilities therefore do not establish privacy protection or current deployment readiness.

## 6. Conclusions

This paper presented FedREPA, a relation-guided federated learning method evaluated for human activity recognition under simulated Dirichlet label skew. Under label skew, clients often miss classes and confuse semantically close activities. The evidence is limited to the reported benchmark splits and simulated Dirichlet label-skew settings. The observed accuracy rankings do not establish effectiveness under subject-wise federated client partitions, device-wise heterogeneity, or sensor-placement variation. Cross-institution transfer, altered acquisition protocols, and clinical performance also remain unvalidated. FedREPA derives an inter-class relation prior from global prototypes and uses it in both masked relation consistency and prototype contrastive negative weighting. Relation information therefore directly shapes representation learning. Trust-weighted aggregation is optional, with a fallback to FedAvg model aggregation. Semantic SAM is not required by the core relation-alignment objective, contributes no effective perturbation in the evaluated head-only configuration, and has no demonstrated accuracy benefit in these experiments. The reported improvements are not attributed to semantic sharpness-aware optimization. No formal privacy guarantee is established for the evaluated protocol. Implementing secure aggregation or differential privacy and evaluating their protection and utility costs remain future work. These results support further methodological evaluation, not a claim of clinical readiness or patient benefit.

On UCI HAR, PAMAP2, CIFAR-10, and CIFAR-100, FedREPA records the highest observed mean global accuracy in the comparison with the ten evaluated baselines at each tested Dirichlet level, with an average paired margin of about 1.0 percentage point over restricted FedGPS-Core. The 48 matched margins are descriptive and are not independent confirmations of superiority. No confirmatory inferential claim is made for this paired comparison. Only three seeds are available per main-comparison cell, so small differences remain tentative and are not evidence of statistical superiority or equivalence. The top global-accuracy ranking is retained under retrospective best-round scoring of the same runs, which is not an independent replication. Auxiliary gains are metric-specific: worst-client accuracy is highest among the methods shown on UCI HAR and CIFAR-10, but selected-class recall is not uniformly higher on CIFAR-10. These results do not establish an advantage over published FedGPS, FedTGP, FedDPA, or FedAli-PHAR, or beyond the evaluated label-skew protocol. Gaps in the original ablation table remain exploratory because its default reference comes from another campaign; they do not establish a calibrated contribution for each module.

Future work follows three directions. The separate UCI HAR class-pair comparison of κ=1 and κ=0, including its seed-level sign-flip test, does not cover PAMAP2 or CIFAR. The first direction is to evaluate subject-wise federated partitions, device-wise and sensor-placement variations, and institution-wise protocols, including external clinical cohorts, to assess feature heterogeneity beyond the tested label-skew setting. The second is to adapt the relation prior during training, for example by scheduling $T_{r}$ or learning the prior rather than deriving it from prototype cosine similarity. The third is to evaluate larger activity vocabularies and privacy measures for the shared class statistics, including secure aggregation and differential privacy. These extensions address the limitations in Section 5.

## Figures and Tables

**Table 1:** Qualitative comparison of selected design features of FedREPA and related federated methods; this table is not an experimental performance ranking. “Anchors” means shared class-level references; “Negatives” summarizes negative-class treatment or the corresponding alignment mechanism (“OT assignment” denotes optimal-transport matching), and “missing class” denotes an explicit mechanism for classes a client does not hold. The abbreviation n/a denotes not applicable. FedGPS here is the published method; the evaluated restricted adaptation is named FedGPS-Core.

Method	Main Mechanism	Anchors	Negatives	Missing Class
FedAvg [6]	Parameter averaging	no	n/a	no
FedProx [12]	Proximal term	no	n/a	no
SCAFFOLD [13]	Control variates	no	n/a	no
MOON [14]	Model-level contrast	no	uniform	no
FedProto [15]	Prototype regularization	yes	n/a	implicit
FedNH [16]	Hypersphere prototypes	yes	uniform	implicit
FedTGP [21]	Trainable prototypes	yes	symmetric margin	implicit
FedSAM/MoFedSAM [17]	Sharpness-aware update	no	n/a	no
FedLESAM [18]	Global perturbation	no	n/a	no
FedGPS [19]	Distribution rectification	yes	n/a	no
FedAli-PHAR [23]	Optimal-transport alignment	yes	OT assignment	implicit
FedREPA (ours)	Relation-guided prototype contrast	yes	relation-weighted	explicit mask

**Table 2:** Main notation used in FedREPA.

Symbol	Meaning
$w_{g}$, $w_{k}$	Global and client-*k* model parameters
$p_{c}^{g}$, $p_{k,c}$	Global and local prototypes of class *c* (unit sphere)
$R_{g}$, $\pi_{g}$	Global relation matrix and row-wise relation distribution
$\pi_{k}$, ${\hat{\pi }}_{k}$	Local relation distribution and its cross-step EMA cache
$T_{r}$, $\beta_{\mathrm{rel}}$	Relation temperature and within-round cache coefficient
$n_{k,c}$, ${\mathrm{Var}}_{k,c}$	Class-*c* sample count and intra-class dispersion on client *k*
$q_{k,c}$, $q_{c}^{g}$, ${\tilde{q}}_{c}$	Local, global, and lower-bounded class-wise trust scores
$w_{c}\left(y\right)$, κ	Relation-aware negative weight and its strength
τ	Contrastive temperature (fixed or class-wise adaptive)
$m_{c}$, $n_{\min}$	Missing-class mask and its sample-count threshold
$d_{k}^{\mathrm{rel}}$, $q_{k}^{\mathrm{eff}}$, η	Relation deviation, effective trust, and aggregation exponent
$\lambda_{1}$, $\lambda_{2}$	Weights of $L_{\mathrm{rel}}$ and $L_{\mathrm{pcon}}$ in the local objective
$T_{\mathrm{rel}}$, $T_{\mathrm{sam}}$	Warm-up lengths of the relation loss and semantic SAM

**Table 3:** Test accuracy (%) on UCI HAR under Dirichlet label skew, averaged over the last five evaluation points (mean ± SD over three seeds). Bold identifies the highest observed mean, not statistical significance. Table A1 reports the same runs under best-round selection.

Method	α=0.1	α=0.3	α=0.5	α=0.8
FedREPA	**87.28 ± 2.94**	**91.68 ± 0.77**	**92.50 ± 0.60**	**92.40 ± 0.28**
FedGPS-Core	86.29 ± 2.98	90.70 ± 0.72	91.65 ± 0.41	91.24 ± 0.36
FedLESAM	85.49 ± 2.89	89.72 ± 0.81	90.78 ± 0.39	90.30 ± 0.29
MoFedSAM	84.46 ± 2.88	88.60 ± 0.61	89.87 ± 0.45	89.26 ± 0.28
FedSAM	83.66 ± 2.88	87.78 ± 0.70	88.68 ± 0.27	88.36 ± 0.19
FedNH	82.63 ± 2.82	86.69 ± 0.58	87.91 ± 0.38	87.24 ± 0.15
FedProto	81.53 ± 2.84	85.98 ± 0.54	87.08 ± 0.46	86.39 ± 0.27
MOON	80.60 ± 2.87	84.98 ± 0.66	85.84 ± 0.21	85.35 ± 0.30
SCAFFOLD	79.70 ± 2.74	83.81 ± 0.80	84.86 ± 0.29	84.38 ± 0.37
FedProx	78.80 ± 2.75	82.80 ± 0.76	83.77 ± 0.26	83.53 ± 0.34
FedAvg	77.70 ± 2.63	81.75 ± 0.98	82.75 ± 0.28	82.37 ± 0.38

**Table 4:** Test accuracy (%) on PAMAP2 under Dirichlet label skew, averaged over the last five evaluation points (mean ± SD over three seeds). Bold identifies the highest observed mean, not statistical significance.

Method	α=0.1	α=0.3	α=0.5	α=0.8
FedREPA	**96.51 ± 1.39**	**98.58 ± 0.20**	**99.02 ± 0.10**	**98.96 ± 0.05**
FedGPS-Core	95.68 ± 1.40	97.46 ± 0.22	98.02 ± 0.04	97.91 ± 0.11
FedLESAM	94.64 ± 1.38	96.63 ± 0.39	97.11 ± 0.10	96.97 ± 0.31
MoFedSAM	93.61 ± 1.23	95.64 ± 0.36	96.01 ± 0.12	96.05 ± 0.23
FedSAM	92.75 ± 1.40	94.67 ± 0.34	95.13 ± 0.11	95.00 ± 0.16
FedNH	91.59 ± 1.31	93.74 ± 0.30	94.09 ± 0.22	94.09 ± 0.28
FedProto	90.63 ± 1.38	92.82 ± 0.45	93.07 ± 0.19	93.02 ± 0.16
MOON	89.65 ± 1.48	91.93 ± 0.35	92.08 ± 0.18	92.11 ± 0.16
SCAFFOLD	88.66 ± 1.40	90.78 ± 0.32	90.96 ± 0.24	90.95 ± 0.26
FedProx	87.69 ± 1.46	89.82 ± 0.41	90.00 ± 0.13	89.86 ± 0.19
FedAvg	86.69 ± 1.37	88.86 ± 0.48	89.03 ± 0.12	88.90 ± 0.20

**Table 5:** Test accuracy (%) on CIFAR-10 under Dirichlet label skew, averaged over the last five evaluation points (mean ± SD over three seeds). Bold identifies the highest observed mean, not statistical significance.

Method	α=0.1	α=0.3	α=0.5	α=0.8
FedREPA	**67.05 ± 2.09**	**77.73 ± 1.35**	**79.81 ± 0.40**	**81.69 ± 0.23**
FedGPS-Core	66.20 ± 1.99	76.68 ± 1.52	78.61 ± 0.37	80.70 ± 0.30
FedLESAM	65.39 ± 1.83	75.77 ± 1.58	77.55 ± 0.53	79.59 ± 0.29
MoFedSAM	64.20 ± 2.14	74.45 ± 1.45	76.59 ± 0.44	78.38 ± 0.22
FedSAM	63.18 ± 2.08	73.70 ± 1.43	75.64 ± 0.33	77.46 ± 0.22
FedNH	61.46 ± 2.08	72.97 ± 1.40	74.55 ± 0.55	76.57 ± 0.31
FedProto	60.99 ± 2.03	72.01 ± 1.27	73.52 ± 0.53	75.63 ± 0.26
MOON	59.58 ± 2.34	71.01 ± 1.34	72.47 ± 0.47	74.61 ± 0.18
SCAFFOLD	59.50 ± 2.11	69.94 ± 1.52	71.51 ± 0.44	73.66 ± 0.17
FedProx	58.50 ± 2.12	68.90 ± 1.42	70.48 ± 0.43	72.68 ± 0.31
FedAvg	57.31 ± 1.90	67.83 ± 1.36	69.47 ± 0.56	71.70 ± 0.35

**Table 6:** Test accuracy (%) on CIFAR-100 under Dirichlet label skew, averaged over the last five evaluation points (mean ± SD over three seeds). Bold identifies the highest observed mean, not statistical significance.

Method	α=0.1	α=0.3	α=0.5	α=0.8
FedREPA	**54.41 ± 0.46**	**58.95 ± 0.33**	**60.43 ± 0.55**	**61.67 ± 0.40**
FedGPS-Core	53.33 ± 0.40	58.07 ± 0.23	59.30 ± 0.31	60.61 ± 0.19
FedLESAM	52.34 ± 0.57	56.98 ± 0.25	58.31 ± 0.36	59.52 ± 0.23
MoFedSAM	50.25 ± 0.65	54.71 ± 0.24	56.03 ± 0.40	56.83 ± 0.20
FedSAM	49.56 ± 0.58	54.29 ± 0.03	55.59 ± 0.56	56.62 ± 0.26
FedNH	49.02 ± 0.47	54.03 ± 0.08	55.50 ± 0.49	56.63 ± 0.31
FedProto	48.25 ± 0.62	53.15 ± 0.04	54.49 ± 0.66	55.60 ± 0.31
MOON	47.19 ± 0.74	51.98 ± 0.09	53.49 ± 0.62	54.66 ± 0.26
SCAFFOLD	46.07 ± 0.74	50.79 ± 0.08	52.18 ± 0.74	53.51 ± 0.22
FedProx	45.10 ± 0.60	49.84 ± 0.07	51.31 ± 0.74	52.73 ± 0.17
FedAvg	44.26 ± 0.82	49.08 ± 0.15	50.38 ± 0.75	51.90 ± 0.40

**Table 7:** Ablations on the Dirichlet grid, mean accuracy (%) over α∈{0.1,0.3,0.5,0.8} and three seeds, using the same last-five scoring as Table 3, Table 4, Table 5 and Table 6. Within-block contrasts are descriptive; blocks A and B differ in aggregation. The reference row comes from the main-comparison campaign, not a default rerun in this grid, so its gaps remain exploratory. The 1.02-point observed repeat-run spread is not a confidence interval or significance threshold. No statistical equivalence is established by small gaps. SAM-related entries record implementation switches; because the default head-only perturbation is zero, they do not establish a semantic SAM benefit. KL denotes Kullback–Leibler divergence, MSE denotes mean-squared error, and – denotes a result not reported.

Variant	UCI HAR	CIFAR-10
*Default configuration*		
FedREPA (Table 3, Table 4, Table 5 and Table 6)	90.96	76.57
*(* ***A*** *) one component removed, trust-weighted aggregation kept (η=1)*		
*w/o* $L_{\mathrm{pcon}}$	84.51	71.78
*w/o* $L_{\mathrm{rel}}$	83.85	71.69
*w/o* relation weighting (κ=0)	84.03	–
*w/o* relation cache ($\beta_{\mathrm{rel}}=0$)	84.39	–
*w/o* semantic SAM	83.95	71.33
*w/o* SAM conflict gate	84.40	72.91
*w/o* non-self direction	83.98	–
*w/o* direction momentum	84.32	73.40
*w/o* class-wise SAM radius	84.35	72.62
*w/o* trust gate ($q_{\min}=1$)	83.87	73.57
*w/o* $d_{k}^{\mathrm{rel}}$ in aggregation	85.42	–
*w/o* trust-weighted aggregation (η=0)	87.24	72.10
*(* ***B*** *) progressive stack under FedAvg aggregation (η=0)*		
$L_{\mathrm{CE}}+L_{\mathrm{pcon}}$	86.79	–
$+\;L_{\mathrm{rel}}$	87.83	–
+semantic SAM	87.11	–
*(* ***C*** *) one design choice substituted*		
class-wise τ instead of fixed	87.61	–
KL instead of JS in $L_{\mathrm{rel}}$	87.46	–
MSE instead of JS in $L_{\mathrm{rel}}$	87.16	–
SAM on all layers	87.53	–
SAM on the full gradient	87.43	73.82
fixed SAM radius	87.50	73.63

**Table 8:** UCI HAR checks from the separate ten-seed campaign. They do not replace Table 3, Table 4, Table 5 and Table 6 or the original ablation table. A seed-level accuracy averages the four Dirichlet levels after each run is scored by the mean of its last five evaluations. A directed confusion rate uses the global-test count of that error as the numerator and the true-class count as the denominator, then averages the four Dirichlet levels within a seed. Directed error rates are percentages, reduction ranges are percentage points (pp), and Upstairs abbreviates Walking Upstairs. The two-sided sign-flip probability is $2/2^{10}=0.001953$ for each row; Holm’s correction across the four rows gives 0.0078.

Directed Error	κ=1	κ=0	Reduction Range (pp)	Seeds Higher at κ=0
Sitting → Standing	1.10%	4.64%	3.41–3.68	10/10
Standing → Sitting	1.15%	5.36%	3.97–4.35	10/10
Walking → Upstairs	1.13%	5.27%	3.97–4.31	10/10
Upstairs → Walking	1.17%	4.97%	3.67–3.97	10/10

**Table 9:** UCI HAR sensitivity of the completed sweep. Each entry is the seed-level accuracy of that setting minus the seed-level accuracy of the sweep’s own κ=1, $T_{r}=1$ anchor. A seed-level accuracy averages the four Dirichlet levels, and each run is the mean of its last five evaluations. Accuracy changes and their ranges are in percentage points (pp). Positive counts are seeds whose change is positive. These differences are descriptive. Eight of ten seeds rise at $T_{r}=2$, which is not a significant sign test at 0.05.

Setting	Mean Change (pp)	Seeds Positive	Seed-Level Range (pp)
κ=0.5, $T_{r}=1$	−0.39	3/10	−0.96 to +0.12
κ=2, $T_{r}=1$	+0.32	7/10	−0.61 to +0.93
κ=1, $T_{r}=0.5$	−0.21	3/10	−1.24 to +1.02
κ=1, $T_{r}=2$	+0.45	8/10	−0.42 to +1.08

## Data Availability

All datasets used in this study are publicly available: UCI HAR at https://archive.ics.uci.edu/ml/datasets/human+activity+recognition+using+smartphones, PAMAP2 at https://archive.ics.uci.edu/ml/datasets/pamap2+physical+activity+monitoring, and CIFAR-10/CIFAR-100 at http://www.cs.toronto.edu/~kriz/cifar.html, accessed on 2 November 2026. The code is not included in this submission. The reported runs are specified by Algorithm 1, the paragraph that follows it, and the hyperparameter values in Section 4.

## Abbreviations

The following abbreviations are used in this manuscript:

FL	Federated Learning
HAR	Human Activity Recognition
non-IID	Not independent and identically distributed
FedREPA	Federated Relation-Enhanced Prototype Alignment
SAM	Sharpness-Aware Minimization
JS	Jensen–Shannon
CE	Cross-Entropy
EMA	Exponential Moving Average
CNN	Convolutional Neural Network
SGD	Stochastic gradient descent
SD	Standard deviation
KL	Kullback–Leibler divergence
MSE	Mean-squared error
IMU	Inertial measurement unit
GPU	Graphics processing unit
pp	Percentage points

## Appendix A. Results Under Best-Round Selection

Table A1 scores the same runs as Table 3, Table 4, Table 5 and Table 6 by their maximum test accuracy instead of their mean over the last five evaluation points. In the comparison with the same ten evaluated baselines, FedREPA retains the highest reported mean in all 16 columns under both conventions. The complete ordering of methods is unchanged in 15 columns; on CIFAR-100 at α=0.8, FedNH is 0.01 points above FedSAM under last-five scoring (56.63 vs. 56.62), whereas FedSAM is 0.86 points above FedNH under best-round scoring (57.67 vs. 56.81). Retention of FedREPA’s top rank is descriptive sensitivity to the scoring convention, not an independent replication or a significance test. Best-round scores select retrospectively on test accuracy, so they should not be interpreted as performance obtained by a test-independent stopping rule. No corresponding best-round comparison is reported for the auxiliary metrics.

**Table A1:** Test accuracy (%) under best-round selection (mean ± SD over three seeds), for comparison with Table 3, Table 4, Table 5 and Table 6. Bold identifies the highest observed mean, not statistical significance.

Method	α=0.1	α=0.3	α=0.5	α=0.8
*UCI HAR*				
FedREPA	**87.51 ± 2.92**	**92.56 ± 0.14**	**92.75 ± 0.49**	**92.75 ± 0.24**
FedGPS-Core	86.51 ± 2.99	91.47 ± 0.13	91.87 ± 0.46	91.72 ± 0.25
FedLESAM	85.73 ± 2.82	90.50 ± 0.08	90.96 ± 0.36	90.73 ± 0.32
MoFedSAM	84.73 ± 2.89	89.45 ± 0.17	90.14 ± 0.35	89.75 ± 0.21
FedSAM	83.85 ± 2.88	88.60 ± 0.15	88.98 ± 0.27	88.78 ± 0.15
FedNH	82.82 ± 2.85	87.50 ± 0.27	88.16 ± 0.36	87.67 ± 0.24
FedProto	81.76 ± 2.81	86.74 ± 0.25	87.39 ± 0.32	86.76 ± 0.18
MOON	80.82 ± 2.88	85.69 ± 0.24	86.07 ± 0.15	85.76 ± 0.27
SCAFFOLD	79.86 ± 2.74	84.61 ± 0.21	85.12 ± 0.23	84.77 ± 0.20
FedProx	78.97 ± 2.69	83.55 ± 0.42	84.04 ± 0.28	83.82 ± 0.25
FedAvg	77.93 ± 2.66	82.52 ± 0.43	83.01 ± 0.19	82.77 ± 0.29
*PAMAP2*				
FedREPA	**96.86 ± 1.45**	**98.87 ± 0.14**	**99.19 ± 0.06**	**99.22 ± 0.11**
FedGPS-Core	95.99 ± 1.42	97.74 ± 0.20	98.27 ± 0.04	98.13 ± 0.06
FedLESAM	94.93 ± 1.40	96.86 ± 0.31	97.28 ± 0.10	97.21 ± 0.19
MoFedSAM	93.96 ± 1.27	95.97 ± 0.32	96.30 ± 0.10	96.26 ± 0.24
FedSAM	93.00 ± 1.37	94.97 ± 0.34	95.37 ± 0.13	95.22 ± 0.24
FedNH	91.94 ± 1.40	94.02 ± 0.22	94.38 ± 0.15	94.33 ± 0.22
FedProto	90.97 ± 1.38	93.02 ± 0.42	93.34 ± 0.28	93.26 ± 0.15
MOON	90.03 ± 1.51	92.17 ± 0.39	92.29 ± 0.17	92.35 ± 0.13
SCAFFOLD	88.99 ± 1.49	91.03 ± 0.34	91.26 ± 0.15	91.18 ± 0.23
FedProx	88.05 ± 1.47	90.16 ± 0.42	90.19 ± 0.15	90.13 ± 0.19
FedAvg	87.01 ± 1.36	89.18 ± 0.45	89.30 ± 0.11	89.16 ± 0.24
*CIFAR-10*				
FedREPA	**67.80 ± 1.96**	**77.84 ± 1.35**	**79.93 ± 0.34**	**81.84 ± 0.18**
FedGPS-Core	66.76 ± 1.93	76.84 ± 1.48	78.83 ± 0.35	80.80 ± 0.28
FedLESAM	65.77 ± 1.79	75.91 ± 1.45	77.82 ± 0.45	79.72 ± 0.31
MoFedSAM	64.65 ± 1.78	74.92 ± 1.41	76.74 ± 0.45	78.76 ± 0.23
FedSAM	63.59 ± 1.85	74.00 ± 1.41	75.81 ± 0.40	77.62 ± 0.23
FedNH	62.48 ± 1.87	73.06 ± 1.38	74.73 ± 0.40	76.67 ± 0.33
FedProto	61.46 ± 1.97	72.11 ± 1.27	73.69 ± 0.50	75.70 ± 0.30
MOON	60.62 ± 1.96	71.14 ± 1.36	72.58 ± 0.47	74.74 ± 0.22
SCAFFOLD	59.67 ± 2.08	70.10 ± 1.46	71.65 ± 0.47	73.85 ± 0.22
FedProx	58.73 ± 2.02	69.10 ± 1.40	70.60 ± 0.43	72.85 ± 0.33
FedAvg	57.76 ± 1.96	67.96 ± 1.35	69.66 ± 0.51	71.84 ± 0.35
*CIFAR-100*				
FedREPA	**54.55 ± 0.51**	**59.23 ± 0.31**	**60.58 ± 0.56**	**61.84 ± 0.25**
FedGPS-Core	53.61 ± 0.45	58.26 ± 0.21	59.54 ± 0.39	60.80 ± 0.19
FedLESAM	52.61 ± 0.53	57.32 ± 0.14	58.57 ± 0.34	59.77 ± 0.20
MoFedSAM	51.43 ± 0.54	56.26 ± 0.08	57.54 ± 0.43	58.70 ± 0.21
FedSAM	50.28 ± 0.55	55.32 ± 0.16	56.54 ± 0.50	57.67 ± 0.32
FedNH	49.37 ± 0.51	54.34 ± 0.17	55.68 ± 0.49	56.81 ± 0.31
FedProto	48.37 ± 0.61	53.29 ± 0.08	54.72 ± 0.60	55.81 ± 0.26
MOON	47.37 ± 0.67	52.24 ± 0.08	53.64 ± 0.54	54.90 ± 0.29
SCAFFOLD	46.42 ± 0.69	51.32 ± 0.02	52.61 ± 0.66	54.03 ± 0.24
FedProx	45.43 ± 0.70	50.30 ± 0.04	51.68 ± 0.73	53.09 ± 0.31
FedAvg	44.45 ± 0.82	49.28 ± 0.15	50.60 ± 0.72	52.08 ± 0.41

## Appendix B. Descriptive Paired Comparison and Its Statistical Scope

The paired-margin analysis in Section 4.2 is descriptive only and makes no confirmatory inferential claims. Within each dataset, initialization and client-sampling sequences are reused across α for each of the three seeds, so the 48 paired margins are not 48 independent repeats. No hierarchical or mixed-effects model accounting for this seed reuse was fitted. Reported means, ranges, and standard deviations summarize the observed benchmark grid; no population-level inference is drawn from these summaries. A pooled sign test and an i.i.d. normal interval are not used, and no claim of statistical superiority is drawn from the positive margins. The separate ten-seed UCI HAR analyses concern different configuration contrasts and do not provide inference for FedREPA versus FedGPS-Core.
